# Hospital and Clinician Practice Variation in Cardiac Surgery and Postoperative Acute Kidney Injury

**DOI:** 10.1001/jamanetworkopen.2025.8342

**Published:** 2025-05-02

**Authors:** Michael R. Mathis, Graciela B. Mentz, Jie Cao, Emily A. Balczewski, Allison M. Janda, Donald S. Likosky, Robert B. Schonberger, Robert B. Hawkins, Michael Heung, Gorav Ailawadi, Rahul Ladhania, Michael W. Sjoding, Sachin Kheterpal, Karandeep Singh

**Affiliations:** 1 Department of Anesthesiology, University of Michigan Medical School, Ann Arbor; 2 Department of Computational Bioinformatics, University of Michigan Medical School, Ann Arbor; 3 Joan and Irwin Jacobs Center for Health Innovation, University of California San Diego; 4 Department of Cardiac Surgery, University of Michigan Medical School, Ann Arbor; 5 Department of Anesthesiology, Yale School of Medicine, New Haven, Connecticut; 6 Nephrology Division, Department of Internal Medicine, University of Michigan Medical School, Ann Arbor; 7 Department of Health Management and Policy, University of Michigan, Ann Arbor; 8 Department of Internal Medicine, Pulmonary and Critical Care Medicine, University of Michigan Medical School, Ann Arbor; 9 Division of Biomedical Informatics, Department of Medicine, University of California, San Diego; 10 Department of Biostatistics, University of Michigan, Ann Arbor

## Abstract

**Importance:**

Approximately 30% of US patients develop acute kidney injury (AKI) after cardiac surgery, which is associated with increased morbidity, mortality, and health care costs. The variation in potentially modifiable hospital- and clinician-level operating room practices and their implications for AKI have not been rigorously evaluated.

**Objective:**

To quantify variation in clinician- and hospital-level hemodynamic and resuscitative practices during cardiac surgery and identify their associations with AKI.

**Design, Setting, and Participants:**

This cohort study analyzed integrated hospital, clinician, and patient data extracted from the Multicenter Perioperative Outcomes Group dataset and the Society of Thoracic Surgeons Adult Cardiac Surgical Database. Participants were adult patients (aged ≥18 years) who underwent cardiac surgical procedures between January 1, 2014, and February 1, 2022, at 8 geographically diverse US hospitals. Patients were followed up through March 2, 2022. Statistical analyses were performed from October 2024 to February 2025.

**Exposures:**

Hospital- and clinician-level variations in operating room hemodynamic practices (inotrope infusion >60 minutes and vasopressor infusion >60 minutes) and resuscitative practices (homologous red blood cell [RBC] transfusion and total fluid volume administration).

**Main Outcomes and Measures:**

The primary outcome was consensus guideline–defined AKI (any stage) within 7 days after cardiac surgery. Hospital- and clinician-level variations were quantified using intraclass correlation coefficients (ICCs). Associations of hospital- and clinician-level practices with AKI were analyzed using multilevel mixed-effects models, adjusting for patient-level characteristics.

**Results:**

Among 23 389 patients (mean [SD] age, 63 [13] years; 16 122 males [68.9%]), 4779 (20.4%) developed AKI after cardiac surgery. AKI rates varied across hospitals (median [IQR], 21.7% [15.5%-27.2%]) and clinicians (18.1% [10.1%-23.7%]). Significant clinician- and hospital-level variation existed for inotrope infusion (ICC, 6.2% [95% CI, 4.2%-8.0%] vs 17.9% [95% CI, 3.3%-31.9%]), vasopressor infusion (ICC, 11.7% [95% CI, 8.3%-14.9%] vs 44.5% [95% CI, 11.7%-63.5%]), RBC transfusion (ICC, 1.7% [95% CI, 0.9%-2.6%] vs 4.5% [95% CI, 1.2%-9.4%]), and fluid volume administration (ICC, 2.1% [95% CI, 1.3%-2.7%] vs 23.8% [95% CI, 2.7%-39.9%]). In multilevel risk-adjusted models, the AKI rate was higher for patients at hospitals with higher inotrope infusion rates (adjusted odds ratio [AOR], 1.98; 95% CI, 1.18-3.33; *P* = .01) and lower among clinicians with higher RBC transfusion rates (AOR, 0.89; 95% CI, 0.79-0.99; *P* = .03). Other practice variations were not associated with AKI.

**Conclusions and Relevance:**

This cohort study of adult patients found that hospital- and clinician-level variation in operating room practices was associated with AKI after cardiac surgery, suggesting possible targets for intervention.

## Key Points

**Question:**

Are specific hospital- and clinician-level operating room practices important factors in interhospital variability in acute kidney injury (AKI) after cardiac surgery?

**Findings:**

In this cohort study involving 23 389 surgical patients in 8 geographically diverse US hospitals, rates of AKI ranged from 11.7% to 32.8%. Adjusted postoperative AKI risk was higher among hospitals more commonly administering inotrope infusions and lower among clinicians more commonly transfusing red blood cells.

**Meaning:**

The findings suggest that interhospital variability in postcardiac surgery AKI is associated with potentially modifiable hospital- and clinician-level operating room practices.

## Teaser

This cohort study assesses operating room practices for vasopressor infusions, inotrope infusions, red blood cell transfusions, and fluid volume administration and their association with postsurgical acute kidney injury in adult patients undergoing cardiac surgery.

## Introduction

Nearly 30% of the 300 000 patients undergoing cardiac surgery annually in the US develop acute kidney injury (AKI).^1,2^ Patients developing AKI after cardiac surgery experience 3 to 4 additional intensive care unit days,^3^ accrue $10 000 added health expenditures,^4^ and incur up to an 8-fold higher odds of mortality.^5^ While 80% to 90% of AKI after cardiac surgery is considered mild, such cases remain associated with substantially increased health care expenditures^6^ and worse outcomes.^7,8^ Given the paucity of evidence-based treatments for AKI once it has developed,^9,10^ most investigations and quality improvement efforts have targeted AKI prevention. While the association of a small set of perioperative practices with preventing AKI has been evaluated via randomized clinical trials and quality initiatives,^11,12,13,14^ most studies focus primarily on postoperative care. The operating room environment is amenable to hemodynamic optimization interventions, which may critically affect AKI, potentially driven by acute blood loss and unique physiological derangements of cardiopulmonary bypass. Yet, the role of a variety of clinical decisions made specifically in the operating room remains unknown.^8,15^ Based on observational studies of patients in the intensive care unit recovering from cardiac surgery, candidate practices relevant to the operating room potentially include inotrope use,^16^ vasopressor use,^17^ homologous red blood cell (RBC) transfusion,^18^ and fluid administration.^19^

Therefore, in this study, we aimed to quantify variations in clinician- and hospital-level hemodynamic and resuscitative practices during cardiac surgery and identify their associations with AKI. Specifically, we evaluated the association between a common set of operating room clinical practices and AKI across a geographically diverse cohort of patients in 8 US hospitals. Furthermore, we quantified the contribution of clinician- vs hospital-level practices to interhospital variability in AKI rates. We tested the hypothesis that variations in prespecified clinician- and hospital-attributable practice preferences (inotropes, vasopressors, transfusions, and fluid administration) during cardiac surgery are associated with AKI outcomes after adjustment for surgical and patient characteristics.

## Methods

### Study Design and Data Source

The University of Michigan Medical School Institutional Review Board approved this cohort study and waived informed consent as the study involved minimal risk to the participants. An a priori study protocol and statistical analysis plan was approved within a peer-review forum and registered prior to analysis (Open Science Framework).^20^ We adhered to the Reporting of Studies Conducted Using Observational Routinely Collected Data (RECORD) reporting guideline.^21^

Data were extracted from the Multicenter Perioperative Outcomes Group dataset and integrated locally with the institutional Society of Thoracic Surgeons Adult Cardiac Surgical Database (STS-ACSD) in 8 hospitals. Methods for local electronic health record (EHR) data acquisition, validation, and secure transfer have been previously described.^22,23^ Similarly, methods for STS-ACSD data acquisition, nurse adjudication, secure transfer, and data quality audits have been described previously.^24^ Patient race and ethnicity characteristics were studied exactly as recorded in the STS-ACSD, abstracted from the EHR by a trained nurse specialist. Race and ethnicity data were collected to evaluate the extent to which (1) study exposures and outcomes varied by racial and ethnic subgroups and (2) study findings were generalizable to other cardiac surgical populations with similar or different racial and ethnic representation.

### Study Population and Primary Outcome

Cardiac surgical procedures in adult patients aged 18 years or older from January 1, 2014, to February 1, 2022, were studied. These procedures included coronary artery bypass, valve repair or replacement, and thoracic aortic procedures in isolation or combination. For patients undergoing multiple procedures, only the index case was used. Additional exclusions are detailed in eFigure 1 in Supplement 1.

The primary outcome was AKI stage 1 or greater as defined by the creatinine component of KDIGO (Kidney Disease: Improving Global Outcomes) international guidelines. Stage 1 is a postoperative increase in serum creatinine level above baseline by at least 0.3 mg/dL within 48 hours or by at least 50% within 7 days.^25^

### Hospital- and Clinician-Level Operating Room Hemodynamic and Resuscitation Practices

Prespecified exposure variables included vasopressor infusions (phenylephrine, norepinephrine, vasopressin, and angiotensin II),^17^ inotrope infusions (epinephrine, milrinone, dobutamine, and dopamine),^16^ RBC transfusions,^18,26^ and fluid volume administration.^19^ Based on previous work,^27^ clinically significant inotrope and vasopressor exposures were defined as infusion durations longer than or equal to 60 minutes intraoperatively. The transfusion exposure was defined as any intraoperative homologous RBC transfusion. The fluid exposure was defined as total crystalloid and colloid administered intraoperatively, excluding blood products, cardiopulmonary bypass priming volume, and ultrafiltration.^28^ Each process of care was studied as a hospital- or clinician-level characteristic, stratified by low (below median) or high (at or above median) rates of use compared with all other clinicians or hospitals in the dataset. Clinicians included the primary attending anesthesiologist (ie, the anesthesiologist attending signed in to a case for the highest number of minutes), the focus of the main analysis, and attending surgeon as the focus of a sensitivity analysis.

### Risk Adjustment 

Preoperative surgical and patient baseline characteristics potentially playing a role in operating room practices and AKI outcomes based on pathophysiological processes and clinical input were considered for risk adjustment (Table 1). Preoperative characteristics were collected from the STS-ACSD. Additional preoperative data from the Multicenter Perioperative Outcomes Group database were collected using validated phenotype algorithms.^29^

**Table 1.** Study Cohort Characteristics

Characteristic	Patients, No. (%)			Standardized differences
	Overall (n = 23 389)	Without AKI (n = 18 610)	With AKI (n = 4779)	
**Demographics**				
Age, y				
18-50	3410 (14.6)	2803 (15.1)	607 (12.7)	0.18
51-60	4965 (21.2)	4041 (21.7)	924 (19.3)	
61-70	7653 (32.7)	6202 (33.3)	1451 (30.4)	
71-80	6071 (26.0)	4648 (25.0)	1423 (29.8)	
≥80	1290 (5.5)	916 (4.9)	374 (7.8)	
Sex				
Male	16 122 (68.9)	12 776 (68.6)	3346 (70.0)	−0.07
Female	7267 (31.1)	5834 (31.4)	1433 (30.0)	
Race				
Asian	484 (2.1)	415 (2.2)	69 (1.4)	0.27
Black	1110 (4.8)	795 (4.3)	315 (6.6)	
White	18 358 (78.5)	14 930 (80.2)	3428 (71.7)	
Multiracial	138 (0.6)	107 (0.6)	31 (0.7)	
Other	614 (2.6)	512 (2.8)	102 (2.1)	
Unknown or declined	2685 (11.5)	1851 (10.0)	834 (17.5)	
Hispanic or Latino ethnicity	799 (3.4)	642 (3.5)	157 (3.3)	−0.06
**Preoperative laboratory values**				
eGFR, mL/min/1.73 m^2^				
≥90: CKD Stage 1	6621 (28.3)	5667 (30.5)	954 (20.0)	0.47
60-89: CKD Stage 2	11 359 (48.6)	9391 (50.5)	1968 (41.2)	
45-59: CKD Stage 3a	3484 (14.9)	2410 (13)	1074 (22.5)	
30-44: CKD Stage 3b	1925 (8.2)	1142 (6.1)	783 (16.4)	
Hemoglobin, g/dL				
>12.0 Females; >13.0 Males: normal	15 082 (64.5)	12 831 (69.0)	2251 (47.1)	0.48
10.1-12.0 Females; 10.1-13.0 Males: mild anemia	6701 (28.7)	4843 (26)	1858 (38.9)	
<10.0: moderate to severe anemia	1606 (6.9)	936 (5.0)	670 (14.0)	
**Medical characteristics**				
ASA Physical Status Classification				
1-3	6780 (29.0)	5893 (31.7)	887 (18.6)	0.34
4	16 274 (69.6)	12 535 (67.4)	3739 (78.2)	
5	335 (1.4)	182 (1.0)	153 (3.2)	
Diabetes				
No	16 798 (71.8)	13 710 (73.7)	3088 (64.6)	0.22
Diagnosed, untreated	1355 (5.8)	1047 (5.6)	308 (6.4)	
Oral hypoglycemics only	3046 (13.0)	2322 (12.5)	724 (15.2)	
Insulin	2190 (9.4)	1531 (8.2)	659 (13.8)	
Hypertension	18 172 (77.7)	14 112 (75.8)	4060 (85.0)	0.23
Heart failure	6958 (29.8)	5073 (27.3)	1885 (39.4)	0.26
Cardiac arrhythmia	5745 (24.6)	4080 (21.9)	1665 (34.8)	0.29
Liver disease	1102 (4.7)	772 (4.2)	330 (6.9)	0.12
Peripheral vascular disease	2737 (11.7)	1906 (10.2)	831 (17.4)	0.21
Cerebrovascular disease	4627 (19.8)	3397 (18.3)	1230 (25.7)	0.18
Preoperative hemodynamics				
Cardiogenic shock, within 24 h	428 (1.8)	238 (1.3)	190 (4.0)	0.17
Ventricular ejection fraction, %				
≥50	18 606 (79.6)	15 140 (81.4)	3466 (72.5)	0.23
40-49	2004 (8.6)	1520 (8.2)	484 (10.1)	
30-39	1215 (5.2)	887 (4.8)	328 (6.9)	
<30	1112 (4.8)	737 (4.0)	375 (7.9)	
Missing data	452 (1.9)	326 (1.8)	126 (2.6)	
Mean arterial pressure, mm Hg				
<70: hypotensive	714 (3.1)	479 (2.6)	235 (4.9)	0.15
108-120: normotensive	3025 (12.9)	2397 (12.9)	628 (13.1)	
70-107: Stage 1 hypertension	17 518 (74.9)	14 123 (75.9)	3395 (71.0)	
>120: Stage 2 hypertension	752 (3.2)	580 (3.1)	172 (3.6)	
Missing data	1380 (5.9)	1031 (5.5)	349 (7.3)	
**Surgical characteristics**				
Surgical procedures, nonmutually exclusive				
Coronary artery bypass	10 154 (43.4)	8118 (43.6)	2036 (42.6)	−0.02
Valve	14 263 (61.0)	11 165 (60.0)	3098 (64.8)	0.10
Aortic	4285 (18.3)	3207 (17.2)	1078 (22.6)	0.13
Previous cardiovascular intervention	8063 (34.5)	5961 (32.0)	2102 (44.0)	0.13
Procedure urgency				
Elective	15 139 (64.7)	12 605 (67.7)	2534 (53.0)	0.35
Urgent	7360 (31.5)	5511 (29.6)	1849 (38.7)	
Emergent	890 (3.8)	494 (2.7)	396 (8.3)	
Cardioplegia delivery				
Antegrade	10 468 (44.8)	8717 (46.8)	1751 (36.6)	0.21
Retrograde	1812 (7.8)	1344 (7.2)	468 (9.8)	
Both	11 109 (47.5)	8549 (45.9)	2560 (53.6)	
Cardiopulmonary bypass duration, mean (SD), h	2.6 (1.3)	2.5 (1.2)	3.1 (1.6)	0.47
Anesthesia duration, mean (SD), h	7.1 (1.9)	6.8 (1.8)	7.9 (2.4)	0.52
Year of procedure				
2014	877 (3.8)	682 (3.7)	195 (4.1)	0.08
2015	1453 (6.2)	1157 (6.2)	296 (6.2)	
2016	1543 (6.6)	1247 (6.7)	296 (6.2)	
2017	3073 (13.1)	2493 (13.4)	580 (12.1)	
2018	4355 (18.6)	3489 (18.8)	866 (18.1)	
2019	4930 (21.1)	3839 (20.6)	1091 (22.8)	
2020	3765 (16.1)	2959 (15.9)	806 (16.9)	
2021	3272 (14.0)	2648 (14.2)	624 (13.1)	
2022	121 (0.5)	96 (0.5)	25 (0.5)	
Anesthesiologist resident present	18 530 (79.2)	14 598 (78.4)	3932 (82.3)	0.10
Certified nurse anesthetist present	259 (1.1)	202 (1.1)	57 (1.2)	0.01
**Anonymized hospitals**				
A	2091 (8.9)	1405 (7.5)	686 (14.4)	0.38
B	4263 (18.2)	3388 (18.2)	875 (18.3)	
C	714 (3.1)	540 (2.9)	174 (3.6)	
D	3446 (14.7)	3044 (16.4)	402 (8.4)	
E	1463 (6.3)	1051 (5.6)	412(8.6)	
F	4540 (19.4)	3866 (20.8)	674 (14.1)	
G	168 (0.7)	139 (0.7)	29 (0.6)	
H	6704 (28.7)	5177 (27.8)	1527 (32.0)	
Operating room practices				
Inotrope infusion, mean (SD), % of total case duration	14.7 (17.3)	12.8 (16.4)	21.8 (18.8)	0.51
Vasopressor infusion, mean (SD), % of total case duration	38.9 (28.1)	37.9 (27.8)	42.9 (28.8)	0.18
Fluid volume administration, mean (SD), mL	2823 (1817)	1826 (1779)	2810 (1960)	−0.01
Homologous RBC transfusion	4441 (19.0)	2891 (15.5)	1550 (32.4)	0.40
Postoperative outcome: 30-d mortality	310 (1.3)	107 (0.6)	203 (4.3)	0.24

Abbreviations: AKI, acute kidney injury; ASA, American Society of Anesthesiologists; CKD, chronic kidney disease; eGFR, estimated glomerular filtration rate; RBC, red blood cell.

### Data Processing

For each variable, missing or outlier values were handled in accordance with registry-specific best practices.^29,30^ Outcome variables were assessed graphically for normality, symmetry, and potential outliers using histograms, Q-Q plots, box plots, and descriptive statistics to inform appropriate modeling strategies. Extreme values were identified using the Tukey-Fences approach and were removed. Given that missing value rates were 5% or lower across all variables meeting inclusion criteria, a complete case analysis was used.

### Statistical Analysis

Statistical analyses were performed from October 2024 to February 2025 using SAS, version 9.4 (SAS Institute Inc), and R, version 4.2.1 (R Project for Statistical Computing). Two-sided *P* < .05 indicated statistical significance.

Distributions of confounders across exposure groups (clinicians and hospitals with low vs high rates of operating room practices) were assessed using standardized differences. Multicollinearity was assessed using Pearson correlation coefficient and variance inflation analyses. In cases of covariate pairs leading to a correlation coefficient greater than 0.50 and variance inflation factor higher than 5, 1 covariate was removed from the multivariable model based on the discretion of the investigators. In cases of collinearity across exposure variables, separate multivariable models were used. Building from this selection process, a final set of parsimonious covariates (patient and surgical characteristics) was identified using the least absolute shrinkage and selection operator for AKI-outcome risk adjustment.

Null generalized linear mixed models were constructed, and modified intraclass correlation coefficient (ICC) estimates were used to assess the relative contributions of each level (patient, clinician, and hospital) to the total variation across operating room practices.^31^ Median odds ratios (ORs) were used to compare the odds of receiving a particular operating room practice for any given patient to hypothetically receive care from 2 randomly selected clinicians or institutions.

Generalized linear mixed models with a logit link function were constructed using low vs high rates of each operating room practice to compute adjusted ORs for developing stage 1 AKI across clinicians nested within hospitals (eMethods in Supplement 1). A hospital- and clinician-level preference-based analytic approach was used to partly mitigate unmeasured confounding affecting both treatment selection and AKI outcomes.

To test the robustness of findings to variations in modeling strategies as well as covariate and outcome definitions, we performed multiple sensitivity analyses. These analyses included (1) the attending surgeon used as the clinician-level nesting variable rather than the attending anesthesiologist; (2) the AKI outcome threshold adjusted to account for more advanced stages (ie, stage ≥2 and stage ≥3)^25^; (3) the AKI outcome with mortality as a competing risk; (4) clinician- and hospital-level high vs low practices as the highest vs lowest quartiles, rather than at or above-median vs below-median practices; (5) a model with the STS-ACSD–derived estimated risk of kidney failure as a covariate in a subset of patients for whom the score was computable; (6) the inotrope exposure modified to include inopressors (epinephrine and dopamine) only; and (7) the inotrope and vasopressor exposures handled as continuous variables (ie, total intraoperative minutes of infusion), with clinician- and hospital-level means computed.

## Results

### Baseline Characteristics

Of the 28 679 cardiac surgical cases reviewed, 23 389 met the inclusion criteria (eFigure 1 in Supplement 1). Clinicians for these cases comprised 198 attending anesthesiologists from 8 hospitals across multiple geographic regions (3 in Northeast, 2 in West, 2 in Midwest, and 1 in South) (eAppendix in Supplement 1). The patient population had a mean (SD) age of 63 (13) years and included 7267 females (31.1%) and 16 122 males (68.9%) (Table 1; eTable 1 in Supplement 2). Nonmutually exclusive cardiac procedures included valve repair or replacement (14 263 [61.0%]), coronary artery bypass (10 154 [43.4%]), and aortic surgery (4285 [18.3%]). Anesthesiology care models most commonly included an attending anesthesiologist supervising an anesthesiology resident (18 530 [79.2%]).

**Figure 1.**
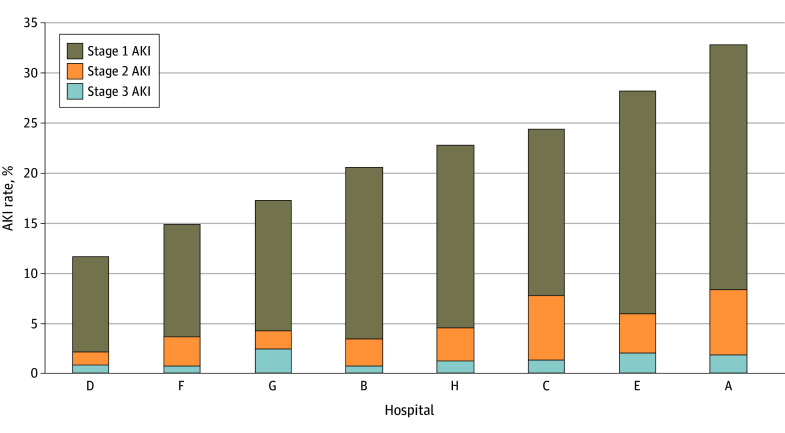
Acute Kidney Injury (AKI) Rates by Hospital

Across the study population, 4779 patients (20.4%) developed AKI stage 1 or greater, including 1008 (4.3%) with stage 2 or greater and 252 (1.1%) with stage 3. Across hospitals, AKI rates ranged from 11.7% to 32.8%, with a median (IQR) of 21.7% (15.5%-27.2%) (Figure 1). Across clinicians, the median (IQR) AKI rate was 18.1% (10.1%-23.7%). Compared with patients without AKI, those with AKI more commonly had comorbidities, underwent urgent or emergent surgical procedures, had longer cardiopulmonary bypass durations, and had lower preoperative estimated glomerular filtration rates and hemoglobin concentrations. All-cause 30-day postoperative mortality rates were 4.9% (203 of 4779) among patients with AKI compared with 0.7% (133 of 19 506) among patients without AKI, for a standardized difference of 0.26.

### Hemodynamic and Resuscitative Practices

Median (IQR) hospital-level practices included inotrope infusions for 16.3% (11.8%-21.0%) of the total case duration, vasopressor infusions for 40.7% (22.8%-58.8%) of the total case duration, fluid volume administration of 2.8 L (2.0-3.3 L), and homologous RBC transfusion for 18.1% (12.7%-19.7%) of cases (eFigure 2 in Supplement 1). Median (IQR) clinician-level practices included inotrope infusions for 14.1% (10.5%-19.5%) of the total case duration, vasopressor infusions for 36.3% (23.9%-53.3%) of the total case duration, fluid volume administration of 3.0 L (2.0-3.6 L), and homologous RBC transfusion for 15.6% (8.8%-21.2%) of cases (eFigure 3 in Supplement 1). Characteristics of cases with low vs high rates of each operating room practice are provided in eTable 2 in Supplement 2.

Combinations of operating room practices across 8 hospitals and 198 clinicians are summarized in Table 2. Within unadjusted models, clinician- vs hospital-level variations existed for each operating room practice, including inotrope infusion (ICC, 6.2% [95% CI, 4.2%-8.0%] vs 17.9% [95% CI, 3.3%-31.9%]), vasopressor infusion (ICC, 11.7% [95% CI, 8.3%-14.9%] vs 44.5% [95% CI, 11.7%-63.5%]), RBC transfusion (ICC, 1.7% [95% CI, 0.9%-2.6%] vs 4.5% [95% CI, 1.2%-9.4%]), and total fluid volume administration (ICC, 2.1% [95% CI, 1.3%-2.7%] vs 23.8% [95% CI, 2.7%-39.9%]) (Table 3).

**Table 2.** Combinations of Hospital- and Clinician-Level Operating Room Practice Patterns

	Inotrope infusion	Vasopressor infusion	RBC transfusion	Total fluid volume administration
**Hospital-level practice patterns**				
Hospitals (n = 8)				
A	High	Low	Low	Low
B	Low	Low	High	Low
C	High	Low	Low	Low
D	Low	Low	Low	High
E	High	High	High	Low
F	Low	High	Low	High
G	High	High	High	High
H	Low	High	High	High
**Clinician-level practice patterns**				
Clinicians, No. (%) (n = 198)				
31 (15.7)	High	Low	Low	Low
27 (13.6)	Low	Low	Low	Low
26 (13.1)	Low	High	High	High
19 (9.6)	High	High	High	High
16 (8.1)	Low	High	Low	High
12 (6.1)	High	High	Low	Low
11 (5.6)	High	Low	High	Low
11 (5.6)	Low	Low	Low	High
10 (5.1)	High	High	Low	High
8 (4.0)	Low	Low	High	High
8 (4.0)	Low	Low	High	Low
7 (3.5)	High	Low	High	High
4 (2.0)	Low	High	Low	Low
3 (1.5)	High	High	High	Low
3 (1.5)	Low	High	High	Low
2 (1.0)	High	Low	Low	High

Abbreviation: RBC, red blood cell.

**Table 3.** Patient-, Clinician-, and Hospital-Attributable Variations in Cardiac Surgery Operating Room Practices

Operating room practice and level	Attributable variation, ICC (95% CI), %	Median OR (95% CI)^a^
Inotrope infustion		
Patient	76.0 (NA)^b^	1 [Reference]
Clinician	6.2 (4.2-8.0)	1.56 (1.43-1.66)
Hospital	17.9 (3.3-31.9)	2.23 (1.37-3.26)
Vasopressor infusion		
Patient	43.8 (NA)^b^	1 [Reference]
Clinician	11.7 (8.3-14.9)	1.87 (1.68-2.05)
Hospital	44.5 (11.7-63.5)	4.68 (1.87-9.68)
RBC transfusion		
Patient	93.8 (NA)^b^	1 [Reference]
Clinician	1.7 (0.9-2.6)	1.26 (1.17-1.32)
Hospital	4.5 (1.2-9.4)	1.45 (1.19-1.74)
Total fluid volume administration		
Patient	74.0 (NA)^b^	1 [Reference]
Clinician	2.1 (1.3-2.7)	NA^c^
Hospital	23.8 (2.7-39.9)	NA^c^

Abbreviations: ICC, intraclass correlation coefficient; NA, not applicable; OR, odds ratio; RBC, red blood cell.

^a^ The median OR is the median value obtained from comparing odds of receiving a particular operating room practice, if the same patient underwent cardiac surgery at 2 different randomly selected hospitals or under the care of 2 different randomly selected clinicians. For example, the value of 2.23 for hospital-level inotrope infusion is interpreted as follows: the median odds of receiving an intraoperative inotrope infusion were more than 2-fold higher if the same patient underwent cardiac surgery at 1 randomly selected hospital vs another.

^b^ The patient level was derived and not part of the model estimates.

^c^ Median ORs not calculable for continuous outcomes.

### Missing Data

Across the study population, 2717 patients (11.6%) had missing data, most commonly data on preoperative mean arterial pressure, left ventricular ejection fraction, and international normalized ratio values. Missing data resulted in a complete case analysis cohort of 20 672 patients for the multilevel models. Compared with cases with complete data, cases with missing data more commonly experienced low vasopressor and fluid administration practices, although such differences did not result in significant changes to the multivariable model cohort (eTable 3 in Supplement 2).

### Multivariable Analyses

**Figure 2.**
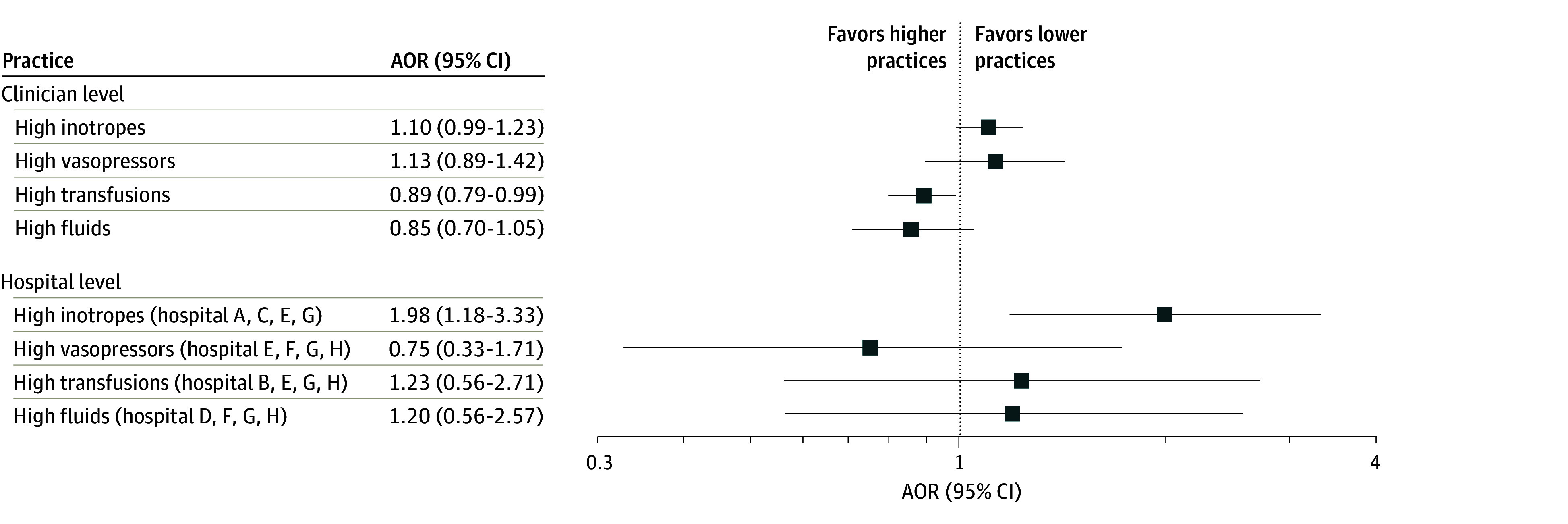
Forest Plots of Clinician- and Hospital-Level Practices and Adjusted Odds Ratio (AOR) of Acute Kidney Injury

Consistent with the a priori statistical analysis plan, a multilevel multivariable modeling approach was used given that more than 5% of the total variance within each practice was explained by hospital and clinician upper levels. Pairwise χ^2^ tests indicated collinearity across practices; therefore, to analyze adjusted associations with the AKI primary outcome, we created separate nested multilevel models for each clinician- and hospital-level practice. A forest plot of hospital- and clinician-level practices and adjusted odds of AKI is shown in Figure 2. Complete models with multivariable selection and adjustment are provided in eTable 4 in Supplement 2.

After adjustment for patient and perioperative characteristics, the odds of AKI were higher for patients at hospitals with high inotrope infusion rates (adjusted OR [AOR], 1.98; 95% CI, 1.18-3.33; *P* = .01) and lower among clinicians with higher RBC transfusion rates (AOR, 0.89; 95% CI, 0.79-0.99; *P* = .03) (Figure 2). Other hospital- and clinician-level practices were not independently associated with increased AKI. Among sensitivity analyses performed, findings were similar to those in the primary analysis (eFigures 4-11 in Supplement 1).

## Discussion

In this multicenter study using integrated EHR and surgical registry data, 20.4% of patients developed AKI after cardiac surgery. This rate, as well as rates for more severe forms of AKI, is consistent with previous reports involving similar cohorts.^32,33^ We observed clinician- and hospital-level variations in both AKI rates and operating room practices plausibly playing a role in AKI, including inotrope infusion, vasopressor infusion, RBC transfusion, and fluid volume administration. Through an analysis of clinician- and hospital-level practice variation, we observed that adjusted rates of AKI were higher across hospitals with higher inotrope infusion rates and lower across clinicians with higher RBC transfusion rates.

Across clinician-level practices, the lower adjusted rates of AKI observed among clinicians with higher RBC transfusion rates should be taken in the context of previous randomized clinical trials examining restrictive vs liberal transfusion strategies.^12,34^ In such trials, restrictive vs liberal transfusion strategies had variable implications for AKI outcomes, although the trials (1) involved standardized transfusion protocols based on serum hemoglobin concentration thresholds, (2) studied AKI as a secondary outcome using varying AKI definitions, and (3) focused on postoperative transfusions. Caution must be taken in the interpretation of our RBC transfusion finding given a CI estimate approaching nonsignificance. However, the finding suggests that optimal operating room transfusion practices for improving AKI outcomes may be different from postoperative transfusion practices, potentially underpinned by acute blood loss during surgery as well as cardiopulmonary bypass–induced pathophysiological processes affecting kidney oxygen delivery and oxidative stress. Furthermore, the results underscore limitations to standardized, threshold-based transfusion strategies potentially failing to incorporate nuanced operating room clinical contexts into transfusion decision-making, which may have been represented in the differences between clinicians with higher vs lower transfusion rates in the operating room. Future investigations of hospital-level intraoperative transfusion practices extending beyond hemoglobin thresholds may include more nuanced goal-directed strategies for improving end-organ perfusion, with additional data sources (eg, cardiopulmonary bypass pump flows), and explore measures of kidney oxygen delivery or myocardial ischemia potentially improved by transfusions,^35,36^ weighed against measures of oxidative stress potentially harmed by transfusions.^37^ Such investigations may also evaluate competing risk outcomes extending beyond AKI (eg, pulmonary complications, health care–associated infections, stroke, and bleeding complications).

Compared with clinician-level practices, hospital-level practices were attributed to larger proportions of total practice variation and had larger effect sizes, including high inotrope infusion rates with an independent association with increased AKI. These findings shed light on the importance of hospital-level practices that are commonly supported by local hospital policies and quality improvement initiatives vs clinician-level practices that are commonly supported by training, experience, and education. Regarding hospital-level variation in inotrope infusions, a study of 29 hospitals (including the 8 in this study) demonstrated a median AOR of 3.55 for a given surgical patient to receive inotropes between 2 randomly selected hospitals, compared with a clinician-level median AOR of 1.73.^27^ This relatively wider hospital-level variation (compared with clinician-level variation) coupled with the large effect size between hospitals with high inotrope infusion rates and associated AKI outcomes may signal opportunities for exploring the impact of hospital-level policies and quality improvement initiatives aimed at promoting patient-centered approaches to inotrope infusion given a lack of benefit from all-or-none approaches.^11^

This study addressed 2 key shortcomings of prior work. First, many of the operating room practices attributed to clinicians may actually be associated with differences in hospital norms, raising the possibility that observed associations may reflect residual confounding from varying practices or referral patterns across hospitals. The association of hospital- and clinician-level variations with patient outcomes has been demonstrated in cardiac surgery^26,27,38^ and is uniquely quantified in the present study, in which we observed the association of hospital-level practice variation with AKI outcomes was relatively more robust compared with clinician-level practice variation. Second, observational studies using EHR data are known to have gaps in data quality that can produce misleading associations in the presence of missing data. These gaps can be partially overcome through integration and adjudication of health data across multiple sources, processes that require substantial resources and expertise to conduct.^22^ This study addresses this issue through the multicenter integration of granular intraoperative EHR data capturing operating room practices into robust cardiac surgical registry data providing improved risk adjustment.

### Limitations

This study has multiple limitations that must be fully considered when interpreting the results. First, although the study benefited from thorough risk adjustment via a multicenter integration of perioperative EHR and surgical registry data, the possibility of residual confounding and biases related to missing data cannot be excluded. This possibility was in part mitigated by studying the associations between clinician- and hospital-level practice preferences and AKI outcomes rather than the patient-level practices themselves. However, external factors affecting such preferences and AKI outcomes potentially existed, for which risk adjustment using the integrated dataset may not have fully accounted. Second, the study analytic plan was limited to hospital- and clinician-attributable variation in 4 prespecified operating room practices; additional operating room or early postoperative practices potentially affecting AKI outcomes may have existed but were beyond the scope of this study. Family-wise error rate adjustment was not used for separate models exploring each practice pattern, given their prespecified selection during study design and favoring an approach of not increasing the chance for a type II error.^39^ Therefore, conclusions about testing a universal null hypothesis (ie, all 4 practice patterns were not simultaneously not associated with AKI) cannot be drawn from this study. Third, the multivariable adjustment we performed was not designed to evaluate associations between practices and competing risks, potentially at odds with AKI-related practices. Fourth, although this study leveraged a geographically diverse set of institutions across the US, community hospitals and hospitals outside the US were not included, potentially limiting the generalizability of the findings.

## Conclusions

Within a multicenter US cohort of patients who underwent cardiac surgery, hospital- and clinician-attributable variation in inotrope infusion and RBC transfusion was found to be associated with AKI. Such variation in hospital- and clinician-level preferences provides insight into candidate strategies that may have implications for AKI and should be explored further in larger studies of intraoperative practice differences across hospitals. These findings suggest a need for future studies investigating optimal patient-centered intraoperative interventions to reduce AKI risk and a need to understand barriers and facilitators to clinician- and hospital-level practice change.

## Notes

Supplement 1. **eAppendix.** Study Hospitals **eMethods.** Statistical Model Design **eFigure 1.** Study Population Inclusion and Exclusion Diagram (Exclusions Applied Sequentially) **eFigure 2.** Caterpillar Plot: Hospital Level **eFigure 3.** Caterpillar Plot: Clinician Level **eFigure 4.** Sensitivity Analysis: Forest Plots of Clinician- and Hospital-Level Practices and Adjusted Odds of Acute Kidney Injury; Surgeons as Nesting Variable **eFigure 5.** Sensitivity Analysis: Forest Plots of Clinician- and Hospital-Level Practices and Adjusted Odds of Acute Kidney Injury; Stage 2 or Greater Acute Kidney Injury **eFigure 6.** Sensitivity Analysis: Forest Plots of Clinician- and Hospital-Level Practices and Adjusted Odds of Acute Kidney Injury; Stage 3 Acute Kidney Injury **eFigure 7.** Sensitivity Analysis: Forest Plots of Clinician- and Hospital-Level Practices and Adjusted Odds of Acute Kidney Injury or Mortality **eFigure 8.** Sensitivity Analysis: Forest Plots of Clinician- and Hospital-Level Practices and Adjusted Odds of Acute Kidney Injury, Comparing Lowest Quartile of Clinicians/Hospitals to Highest Quartile **eFigure 9.** Sensitivity Analysis: Forest Plots of Clinician- and Hospital-Level Practices and Adjusted Odds of Acute Kidney Injury, Including the Society of Thoracic Surgeons Predicted Risk of Renal Failure as a Covariate in the Subset of Patients for Whom the Score was Computable (n = 12,970) **eFigure 10.** Sensitivity Analysis: Forest Plots of Clinician- and Hospital-Level Practices and Adjusted Odds of Acute Kidney Injury, Considering Inopressors Only (Epinephrine, Dopamine) **eFigure 11.** Sensitivity Analysis: Forest Plots of Clinician- and Hospital-Level Practices and Adjusted Odds of Acute Kidney Injury, Considering Inotrope and Vasopressor Exposures Based upon Total Intraoperative Infusion Duration

Supplement 2. **eTable 1.** Study Cohort Extended Characteristics **eTable 2.** Study Cohort Characteristics by Clinician- and Hospital-Level Operating Room Practices **eTable 3.** Missing Data Analysis **eTable 4.** Multivariable Models for Clinician- and Hospital-Level Operating Room Practices and Postoperative Acute Kidney Injury

Supplement 3.
Nonauthor Collaborators

Supplement 4.
Data Sharing Statement
