# Supplementary material for: Hospital and Clinician Practice Variation in Cardiac Surgery and Postoperative Acute Kidney Injury
Source: JAMA Netw Open. 2025 May 2;8(5):e258342. doi: 10.1001/jamanetworkopen.2025.8342 (PMC12048843; doi:10.1001/jamanetworkopen.2025.8342)
Supplement: Supplement 1. — eAppendix. Study Hospitals eMethods. Statistical Model Design eFigure 1. Study Population Inclusion and Exclusion Diagram (Exclusions Applied Sequentially) eFigure 2. Caterpillar Plot: Hospital Level eFigure 3. Caterpillar Plot: Clinician Level eFigure 4. Sensitivity Analysis: Forest Plots of Clinician- and Hospital-Level Practices and Adjusted Odds of Acute Kidney Injury; Surgeons as Nesting Variable eFigure 5. Sensitivity Analysis: Forest Plots of Clinician- and Hospital-Level Practices and Adjusted Odds of Acute Kidney Injury; Stage 2 or Greater Acute Kidney Injury eFigure 6. Sensitivity Analysis: Forest Plots of Clinician- and Hospital-Level Practices and Adjusted Odds of Acute Kidney Injury; Stage 3 Acute Kidney Injury eFigure 7. Sensitivity Analysis: Forest Plots of Clinician- and Hospital-Level Practices and Adjusted Odds of Acute Kidney Injury or Mortality eFigure 8. Sensitivity Analysis: Forest Plots of Clinician- and Hospital-Level Practices and Adjusted Odds of Acute Kidney Injury, Comparing Lowest Quartile of Clinicians/Hospitals to Highest Quartile eFigure 9. Sensitivity Analysis: Forest Plots of Clinician- and Hospital-Level Practices and Adjusted Odds of Acute Kidney Injury, Including the Society of Thoracic Surgeons Predicted Risk of Renal Failure as a Covariate in the Subset of Patients for Whom the Score was Computable (n = 12,970) eFigure 10. Sensitivity Analysis: Forest Plots of Clinician- and Hospital-Level Practices and Adjusted Odds of Acute Kidney Injury, Considering Inopressors Only (Epinephrine, Dopamine) eFigure 11. Sensitivity Analysis: Forest Plots of Clinician- and Hospital-Level Practices and Adjusted Odds of Acute Kidney Injury, Considering Inotrope and Vasopressor Exposures Based upon Total Intraoperative Infusion Duration [file jamanetwopen-e258342-s001.pdf]

Supplemental Online Content

Mathis MR, Mentz GB, Cao J, et al; the MPOG Collaborators. Hospital and clinician practice variation in cardiac surgery and postoperative acute kidney injury. *JAMA Netw Open*. 2025;8(5):e258342. doi:10.1001/jamanetworkopen.2025.8342

eAppendix. Study Hospitals
eMethods. Statistical Model Design
eFigure 1. Study Population Inclusion and Exclusion Diagram (Exclusions Applied Sequentially)
eFigure 2. Caterpillar Plot: Hospital Level
eFigure 3. Caterpillar Plot: Clinician Level
eFigure 4. Sensitivity Analysis: Forest Plots of Clinician- and Hospital-Level Practices and Adjusted Odds of Acute Kidney Injury; Surgeons as Nesting Variable
eFigure 5. Sensitivity Analysis: Forest Plots of Clinician- and Hospital-Level Practices and Adjusted Odds of Acute Kidney Injury; Stage 2 or Greater Acute Kidney Injury
eFigure 6. Sensitivity Analysis: Forest Plots of Clinician- and Hospital-Level Practices and Adjusted Odds of Acute Kidney Injury; Stage 3 Acute Kidney Injury
eFigure 7. Sensitivity Analysis: Forest Plots of Clinician- and Hospital-Level Practices and Adjusted Odds of Acute Kidney Injury or Mortality
eFigure 8. Sensitivity Analysis: Forest Plots of Clinician- and Hospital-Level Practices and Adjusted Odds of Acute Kidney Injury, Comparing Lowest Quartile of Clinicians/Hospitals to Highest Quartile
eFigure 9. Sensitivity Analysis: Forest Plots of Clinician- and Hospital-Level Practices and Adjusted Odds of Acute Kidney Injury, Including the Society of Thoracic Surgeons Predicted Risk of Renal Failure as a Covariate in the Subset of Patients for Whom the Score was Computable (n = 12,970)
eFigure 10. Sensitivity Analysis: Forest Plots of Clinician- and Hospital-Level Practices and Adjusted Odds of Acute Kidney Injury, Considering Inopressors Only (Epinephrine, Dopamine)
eFigure 11. Sensitivity Analysis: Forest Plots of Clinician- and Hospital-Level Practices and Adjusted Odds of Acute Kidney Injury, Considering Inotrope and Vasopressor Exposures Based upon Total Intraoperative Infusion Duration

This supplemental material has been provided by the authors to give readers additional information about their work.

© 2025 Mathis MR et al. *JAMA Network Open*

eAppendix. Study Hospitals

Brigham and Women's Hospital, Boston, Massachusetts

Massachusetts General Hospital, Boston, Massachusetts

Oregon Health and Science University, Portland, Oregon

University of Michigan Health System, Ann Arbor, Michigan

University of Virginia Health System, Charlottesville, Virginia

University of Washington Medical Center, Seattle, Washington

Washington University of St. Louis School of Medicine, St. Louis, Missouri

Yale New Haven Hospital, New Haven, Connecticut

© 2025 Mathis MR et al. *JAMA Network Open*

eMethods. Statistical Model Design

Separate models individually considering each practice pattern (inotrope use, vasopressor use, homologous red blood cell transfusion, or fluid administration) were:

<u>Patient Level</u>: $(AKI)_{ijk} = \beta_{0jk}$ + (Patient-level covariates)

<u>Clinician Level</u>: $\beta_{0jk} = \delta_{0k} + \delta_{1k}$ * (*Clinician Practice Pattern*) + error

<u>Hospital Level</u>: $\delta_{0k} = \gamma_{00} + \gamma_{01}$* (*Hospital Practice Pattern*) + error

$\delta_{1k} = \gamma_{10} + \gamma_{11}$* (*Hospital Practice Pattern*) + error

where *i* = 1,2,…I patients; *j* = 1,2…J clinicians and *k* = 1,2…K hospitals

Where *Clinician Practice Pattern* is a binary indicator taking the value of 0 for <u>clinician-level</u> practice pattern rates below the median and 1 for those at or above the median; and where *Hospital Practice Pattern* is a binary indicator taking the value of 0 for <u>hospital-level</u> rates below the median and 1 for those at or above the median.

© 2025 Mathis MR et al. *JAMA Network Open*

eFigure 1. Study Population Inclusion and Exclusion Diagram (exclusions applied sequentially)

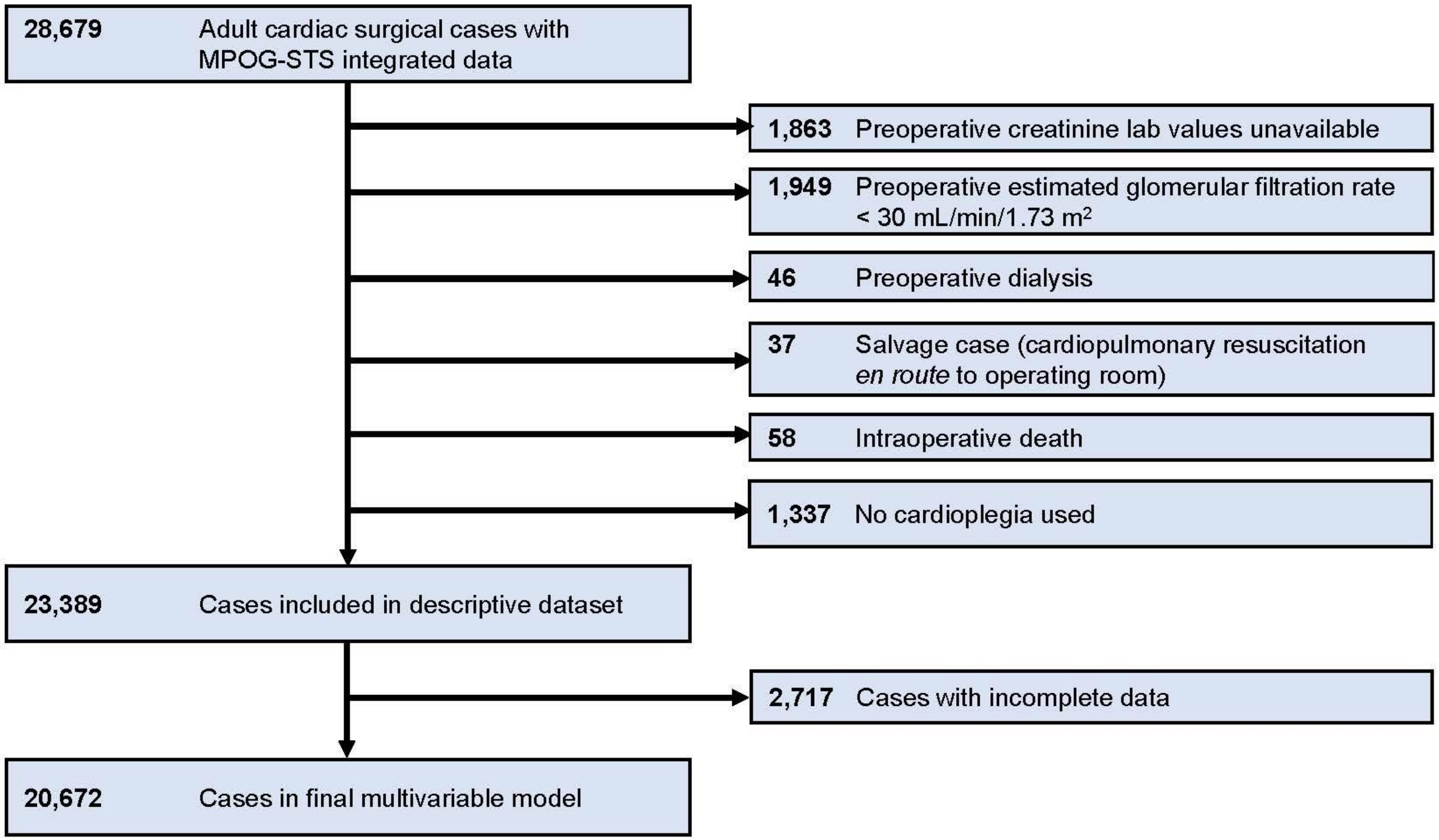

© 2025 Mathis MR et al. *JAMA Network Open*

eFigure 2. Caterpillar Plot: Hospital Level

A. Hospital-level Inotrope Infusion Use

Hospital	Lower Bound	Mean	Upper Bound
F	10.6	11.1	11.5
D	10.9	11.4	12.0
B	12.6	13.1	13.6
H	13.0	13.4	13.9
C	18.1	19.2	20.4
G	16.7	19.3	21.8
E	20.8	21.6	22.4
A	27.5	28.1	28.8

© 2025 Mathis MR et al. *JAMA Network Open*

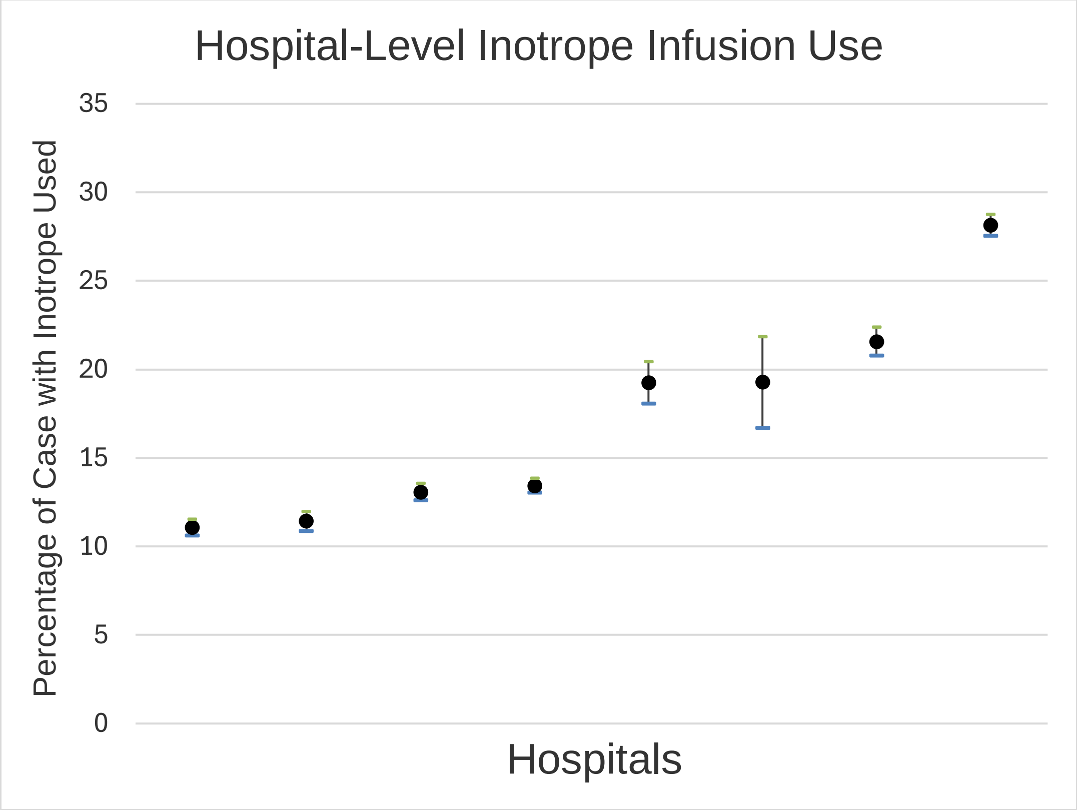

© 2025 Mathis MR et al. *JAMA Network Open*

B. Hospital-level Vasopressor Infusion Use

Hospital	Lower Bound	Mean	Upper Bound
B	13.2	13.9	14.5
A	20.2	21.0	21.8
D	27.5	28.2	28.9
F	36.7	37.2	37.8
C	42.3	44.2	46.1
G	53.8	56.7	59.6
E	58.2	59.5	60.7
H	61.1	61.6	62.2

© 2025 Mathis MR et al. *JAMA Network Open*

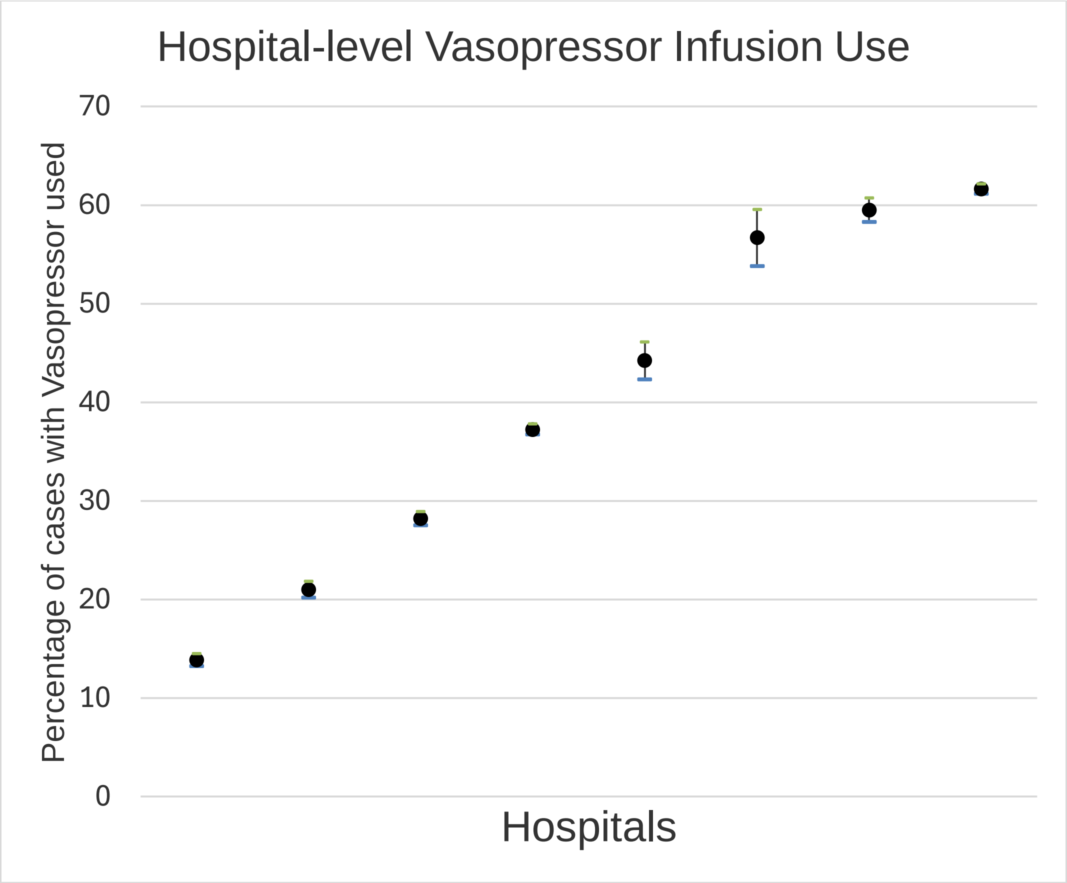

© 2025 Mathis MR et al. *JAMA Network Open*

C. Hospital-level Homologous Red Blood Cell Transfusion Use

Hospital	Lower Bound	Mean	Upper Bound
A	6.4	7.6	8.7
C	9.7	12.0	14.4
D	13.4	14.6	15.7
F	16.3	17.4	18.5
B	17.6	18.8	20.0
G	13.6	19.6	25.7
H	18.8	19.7	20.7
E	48.8	51.3	53.9

© 2025 Mathis MR et al. *JAMA Network Open*

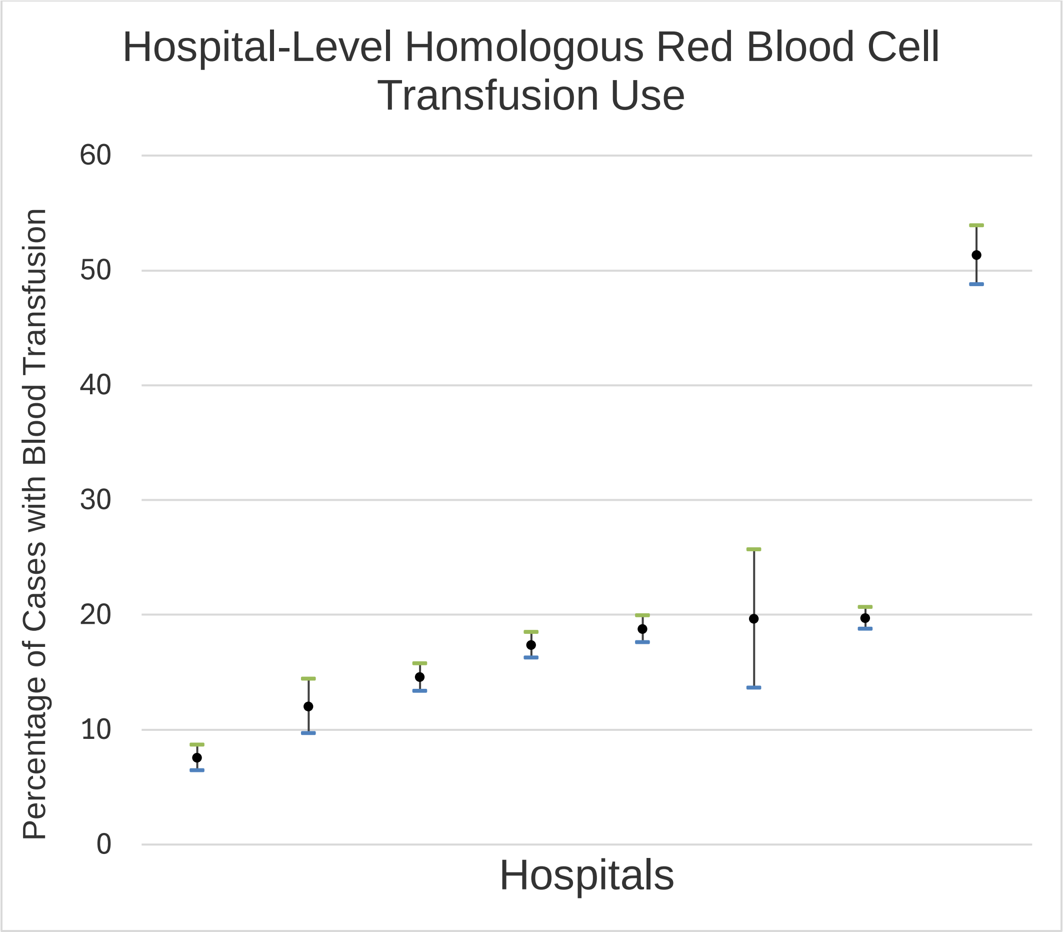

© 2025 Mathis MR et al. *JAMA Network Open*

D. Hospital-level Total Fluid Administration

Hospital	Lower Bound	Mean	Upper Bound
B	1302	1341	1380
A	1754	1801	1848
C	2547	2636	2726
E	2567	2672	2778
D	2844	2888	2932
G	2746	3002	3259
H	3313	3352	3392
F	3870	3925	3980

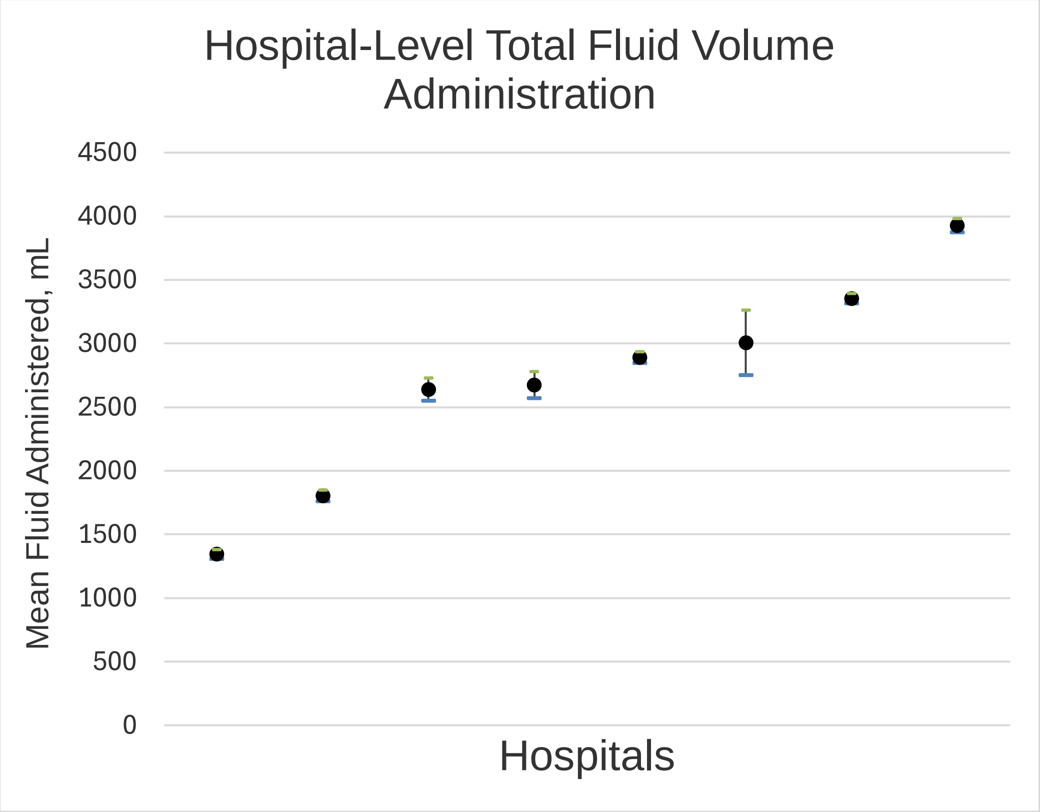

© 2025 Mathis MR et al. *JAMA Network Open*

eFigure 3. Caterpillar Plot: Clinician Level

A. Clinician-Level Inotrope Infusion Use

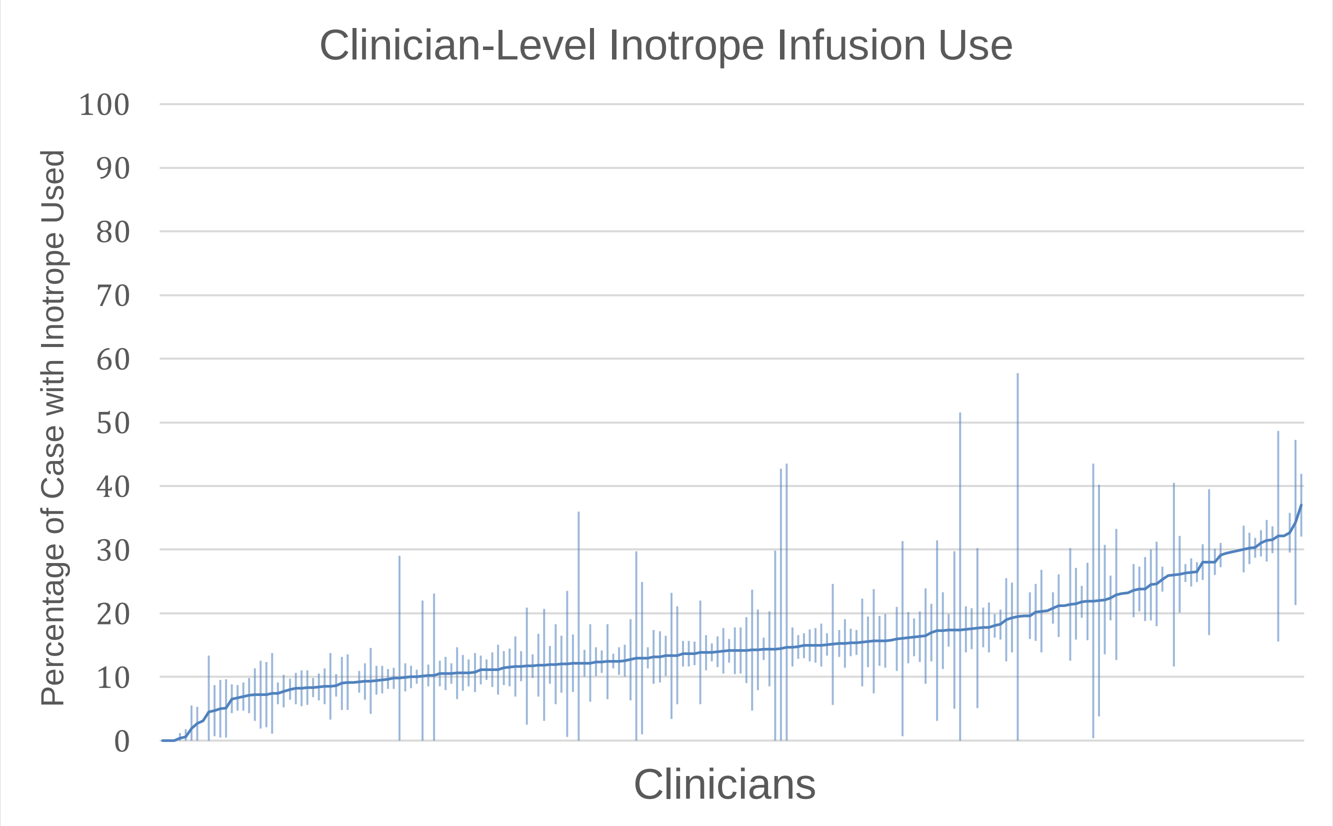

© 2025 Mathis MR et al. *JAMA Network Open*

B. Clinician-Level Vasopressor Infusion Use

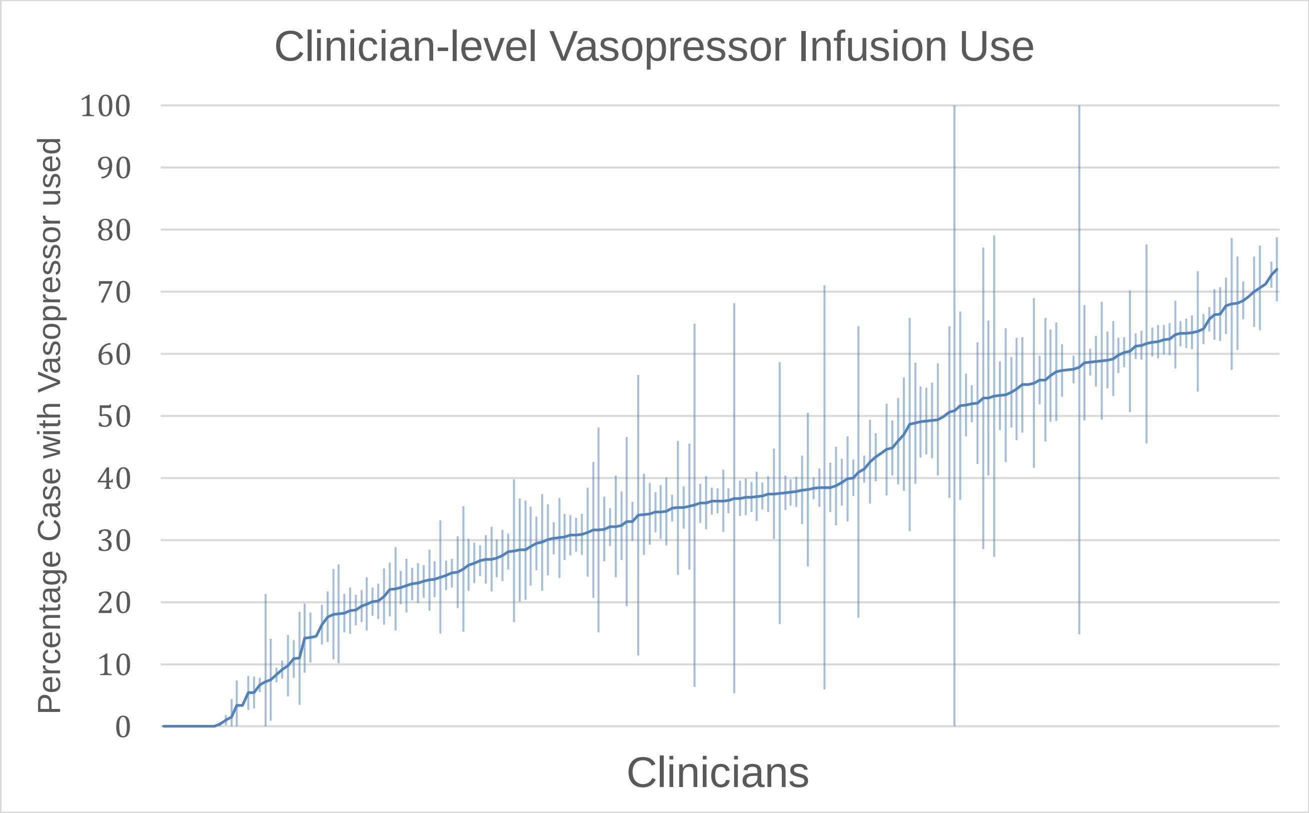

© 2025 Mathis MR et al. *JAMA Network Open*

C. Clinician-level Homologous Red Blood Cell Transfusion Use

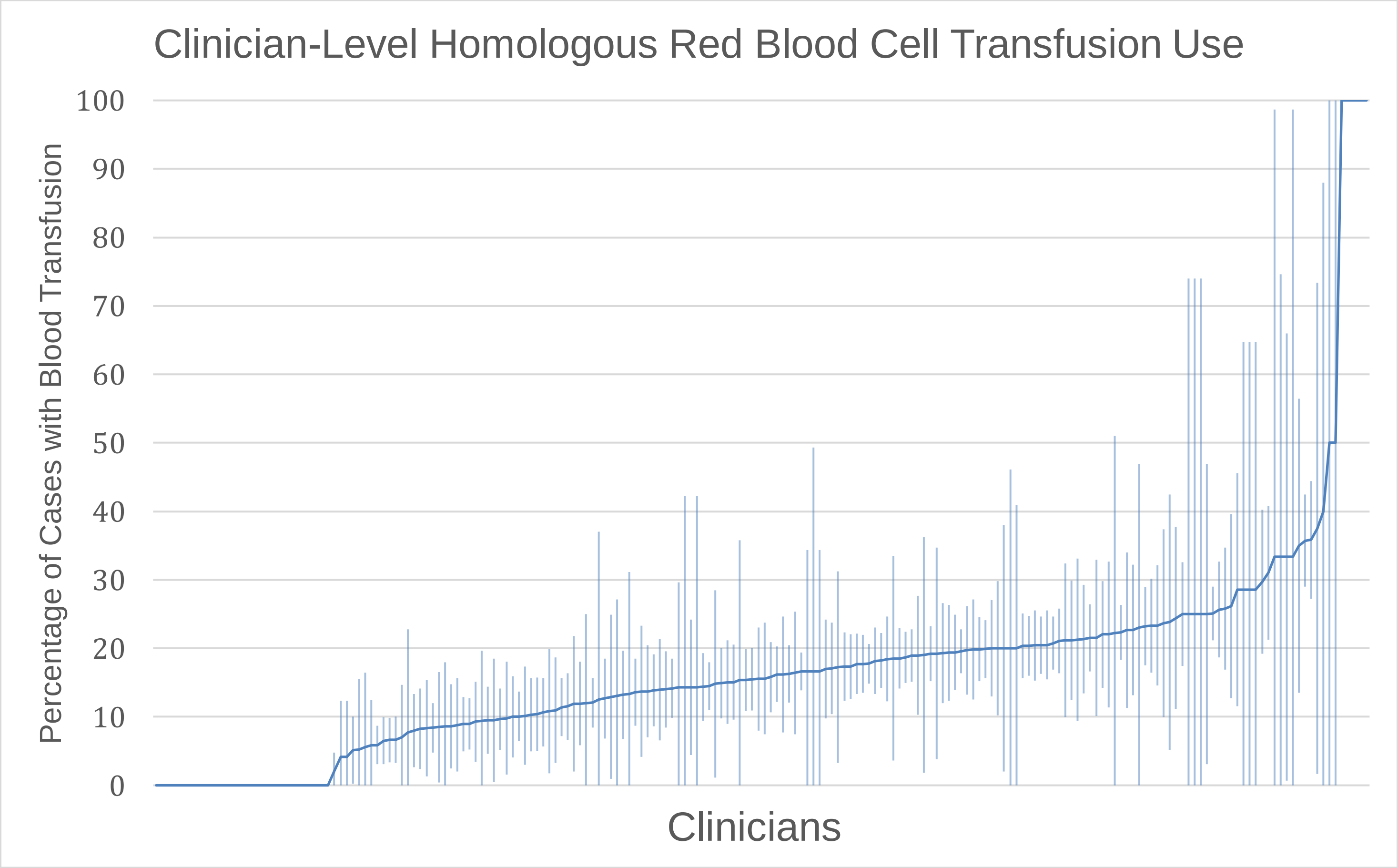

© 2025 Mathis MR et al. *JAMA Network Open*

D. Clinician-level Total Fluid Administration

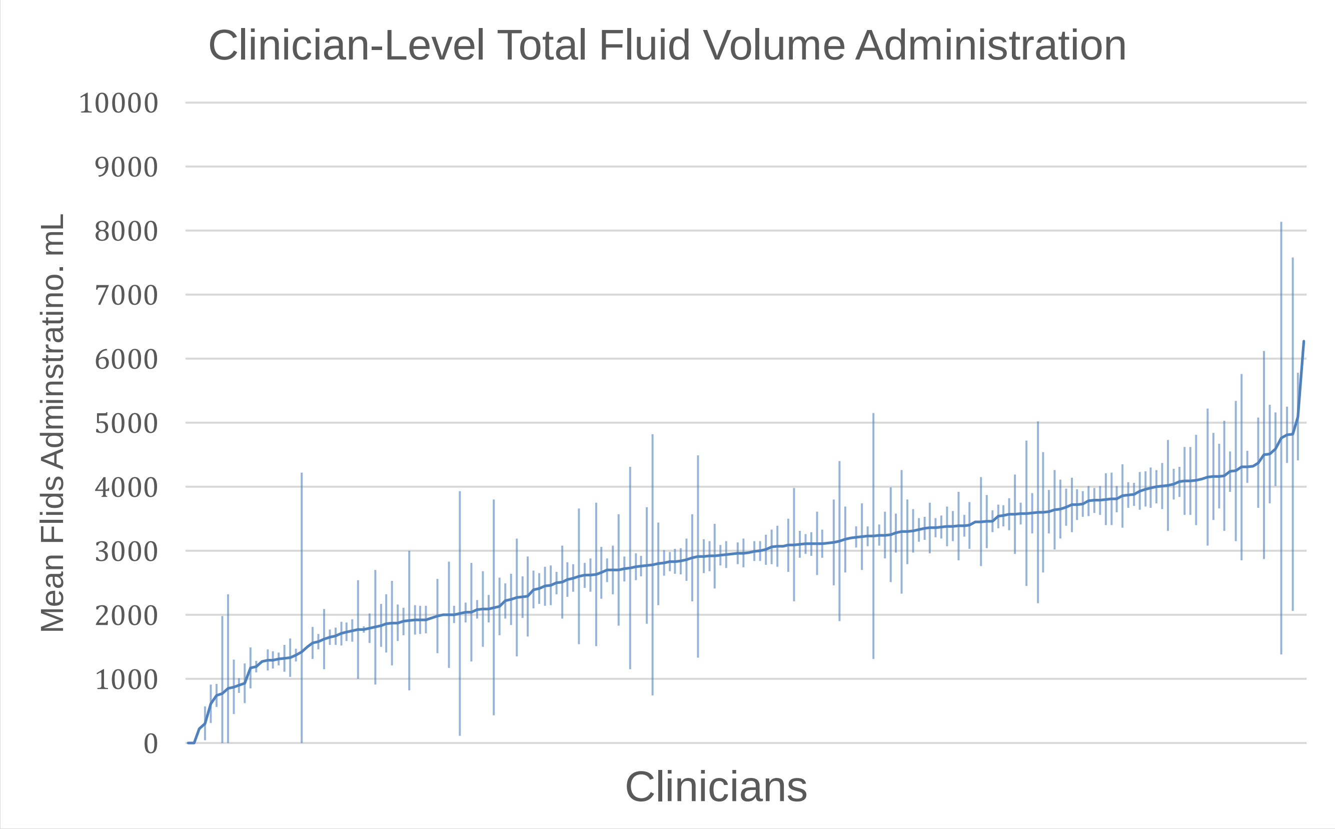

© 2025 Mathis MR et al. *JAMA Network Open*

eFigure 4. Sensitivity Analysis: Forest Plots of Clinician- and Hospital-Level Practices and Adjusted Odds of Acute Kidney Injury; Surgeons as Nesting Variable

	Practice	OR (95% CI)
Clinician Level Practices	High Inotropes	1.13 (1.01, 1.26)
	High Vasopressors	1.20 (0.95, 1.52)
	High Transfusions	0.93 (0.83, 1.05)
	High Fluids	0.93 (0.76, 1.12)
Hospital Level Practices	High Inotropes (A, C, E, G)	1.52 (0.92, 2.51)
	High Vasopressors (E, F, G, H)	0.75 (0.39, 1.43)
	High Transfusions (B, E, G, H)	1.20 (0.63, 2.26)
	High Fluids (D, F, G, H)	1.18 (0.63, 2.21)

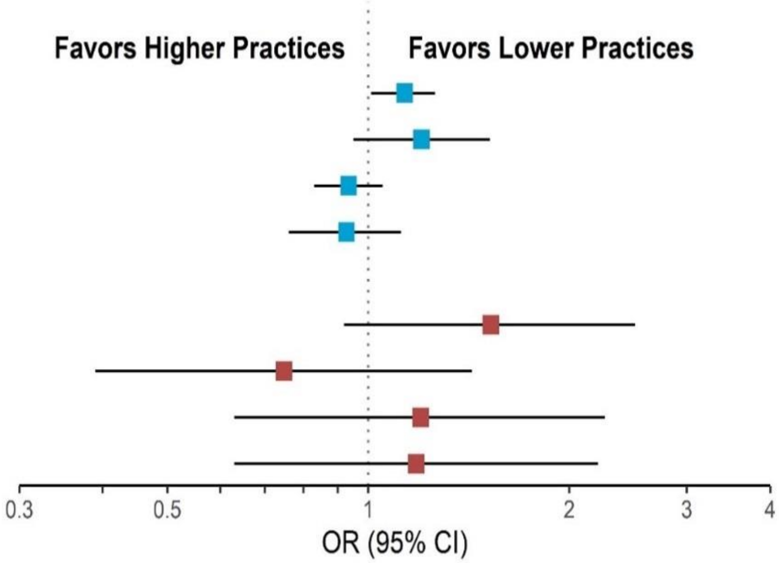

Hospital- and clinician-level operating room practices were defined as high (at or above median) or low (below median) rates of inotrope infusions (epinephrine, milrinone, dobutamine, or dopamine for >60 minutes), vasopressor infusions (phenylephrine, norepinephrine, vasopressin, or angiotensin-II for >60 minutes), homologous red blood cell transfusion, and fluid volume administration (total crystalloid and colloid administered, excluding blood products). Anonymized hospital IDs were provided for the with each "high" practice.

© 2025 Mathis MR et al. *JAMA Network Open*

eFigure 5. Sensitivity Analysis: Forest Plots of Clinician- and Hospital-Level Practices and Adjusted Odds of Acute Kidney Injury; Stage 2 or Greater Acute Kidney Injury

	Practice	OR (95% CI)
Clinician Level Practices	High Inotropes	1.18 (0.97, 1.43)
	High Vasopressors	1.44 (0.96, 2.18)
	High Transfusions	0.82 (0.67, 1.00)
	High Fluids	0.87 (0.61, 1.24)
Hospital Level Practices	High Inotropes (A, C, E, G)	2.30 (1.50, 3.55)
	High Vasopressors (E, F, G, H)	0.59 (0.23, 1.51)
	High Transfusions (B, E, G, H)	0.97 (0.42, 2.27)
	High Fluids (D, F, G, H)	0.90 (0.38, 2.13)

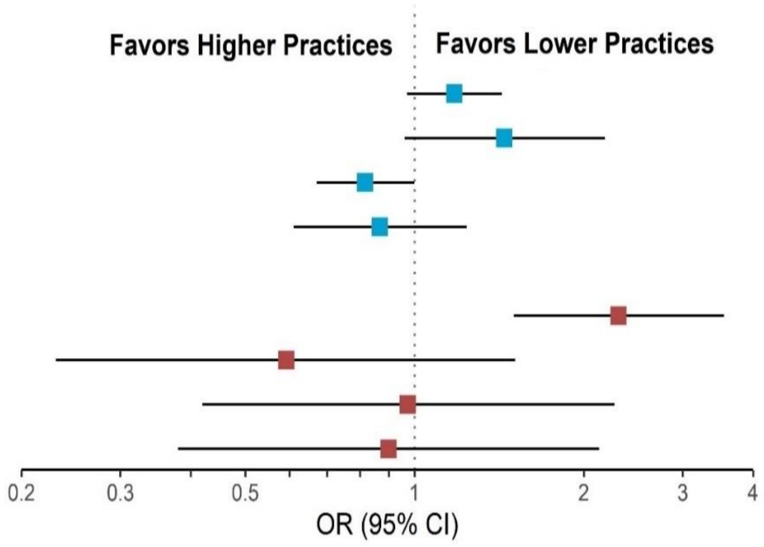

Hospital- and clinician-level operating room practices were defined as high (at or above median) or low (below median) rates of inotrope infusions (epinephrine, milrinone, dobutamine, or dopamine for >60 minutes), vasopressor infusions (phenylephrine, norepinephrine, vasopressin, or angiotensin-II for >60 minutes), homologous red blood cell transfusion, and fluid volume administration (total crystalloid and colloid administered, excluding blood products). Anonymized hospital IDs were provided for the with each "high" practice.

© 2025 Mathis MR et al. *JAMA Network Open*

eFigure 6. Sensitivity Analysis: Forest Plots of Clinician- and Hospital-Level Practices and Adjusted Odds of Acute Kidney Injury; Stage 3 Acute Kidney Injury

	Practice	OR (95% CI)
Clinician Level Practices	High Inotropes	1.05 (0.72, 1.54)
	High Vasopressors	1.51 (0.64, 3.56)
	High Transfusions	0.76 (0.51, 1.14)
	High Fluids	0.54 (0.33, 0.90)
Hospital Level Practices	High Inotropes (A, C, E, G)	2.49 (1.53, 4.07)
	High Vasopressors (E, F, G, H)	0.50 (0.17, 1.47)
	High Transfusions (B, E, G, H)	1.11 (0.49, 2.52)
	High Fluids (D, F, G, H)	1.01 (0.52, 1.97)

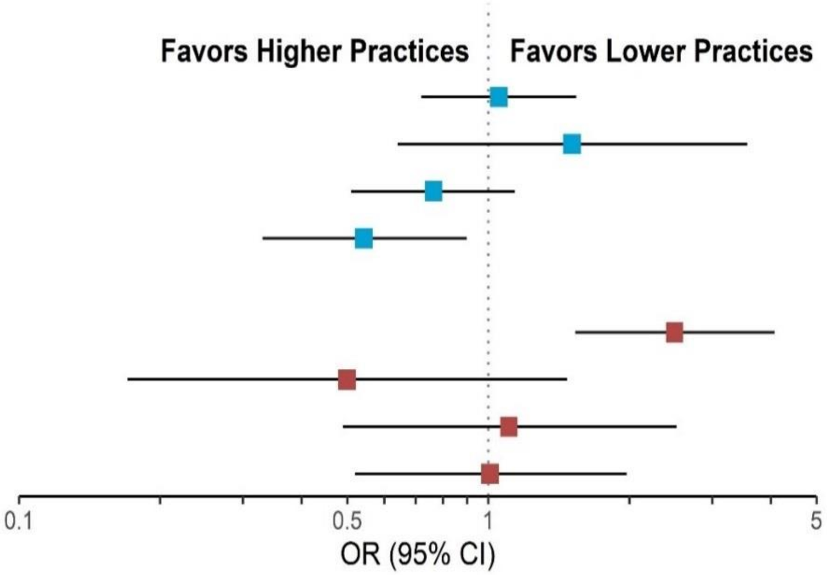

Hospital- and clinician-level operating room practices were defined as high (at or above median) or low (below median) rates of inotrope infusions (epinephrine, milrinone, dobutamine, or dopamine for >60 minutes), vasopressor infusions (phenylephrine, norepinephrine, vasopressin, or angiotensin-II for >60 minutes), homologous red blood cell transfusion, and fluid volume administration (total crystalloid and colloid administered, excluding blood products). Anonymized hospital IDs were provided for the with each "high" practice.

© 2025 Mathis MR et al. *JAMA Network Open*

eFigure 7. Sensitivity Analysis: Forest Plots of Clinician- and Hospital-Level Practices and Adjusted Odds of Acute Kidney Injury or Mortality

	Practice	OR (95% CI)
Clinician Level Practices	High Inotropes	1.10 (0.99, 1.22)
	High Vasopressors	1.11 (0.88, 1.39)
	High Transfusions	0.89 (0.80, 0.99)
	High Fluids	0.87 (0.71, 1.06)
Hospital Level Practices	High Inotropes (A, C, E, G)	1.95 (1.18, 3.22)
	High Vasopressors (E, F, G, H)	0.75 (0.34, 1.67)
	High Transfusions (B, E, G, H)	1.22 (0.57, 2.60)
	High Fluids (D, F, G, H)	1.18 (0.56, 2.48)

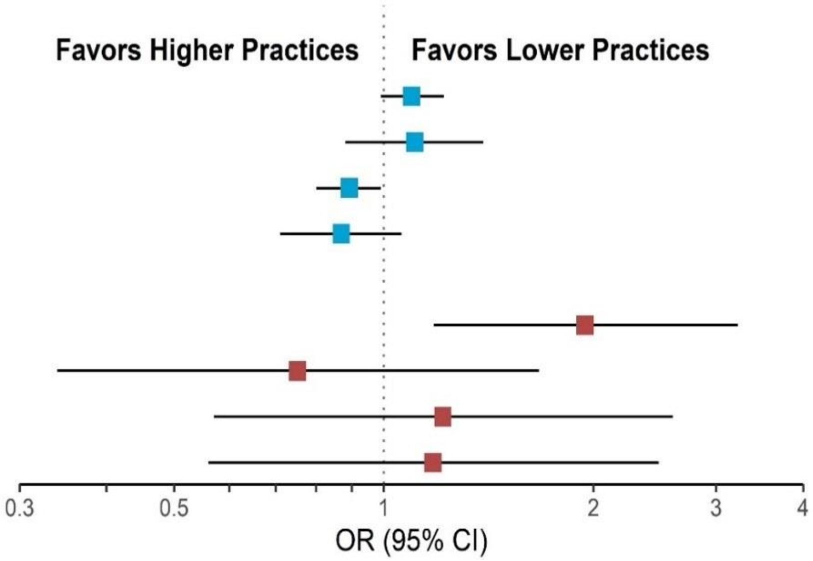

Hospital- and clinician-level operating room practices were defined as high (at or above median) or low (below median) rates of inotrope infusions (epinephrine, milrinone, dobutamine, or dopamine for >60 minutes), vasopressor infusions (phenylephrine, norepinephrine, vasopressin, or angiotensin-II for >60 minutes), homologous red blood cell transfusion, and fluid volume administration (total crystalloid and colloid administered, excluding blood products). Anonymized hospital IDs were provided for the with each "high" practice.

© 2025 Mathis MR et al. *JAMA Network Open*

eFigure 8. Sensitivity Analysis: Forest Plots of Clinician- and Hospital-Level Practices and Adjusted Odds of Acute Kidney Injury, Comparing Lowest Quartile of Clinicians / Hospitals to Highest Quartile

	Practice	OR (95% CI)
Clinician Level Practices	High Inotropes	0.95 (0.72, 1.26)
	High Vasopressors	1.08 (0.93, 1.26)
	High Transfusions	1.02 (0.89, 1.17)
	High Fluids	0.96 (0.79, 1.16)
Hospital Level Practices	High Inotropes (A, C, E, G)	2.57 (1.07, 6.13)
	High Vasopressors (E, F, G, H)	1.08 (0.43, 2.68)
	High Transfusions (B, E, G, H)	0.87 (0.28, 2.73)
	High Fluids (D, F, G, H)	0.67 (0.29, 1.52)

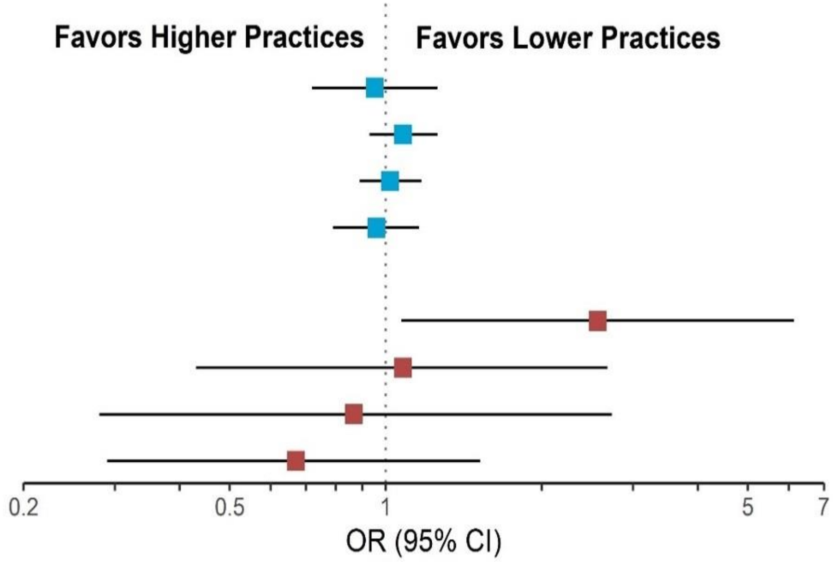

Hospital- and clinician-level operating room practices were defined as high (at or above median) or low (below median) rates of inotrope infusions (epinephrine, milrinone, dobutamine, or dopamine for >60 minutes), vasopressor infusions (phenylephrine, norepinephrine, vasopressin, or angiotensin-II for >60 minutes), homologous red blood cell transfusion, and fluid volume administration (total crystalloid and colloid administered, excluding blood products). Anonymized hospital IDs were provided for the with each "high" practice.

© 2025 Mathis MR et al. *JAMA Network Open*

eFigure 9. Sensitivity Analysis: Forest Plots of Clinician- and Hospital-Level Practices and Adjusted Odds of Acute Kidney Injury, Including the Society of Thoracic Surgeons Predicted Risk of Renal Failure as a Covariate in the Subset of Patients for Whom the Score was Computable (n = 12,970)

	Practice	OR (95% CI)
Clinician Level Practices	High Inotropes	1.03 (0.90, 1.19)
	High Vasopressors	1.03 (0.78, 1.37)
	High Transfusions	0.86 (0.75, 0.98)
	High Fluids	0.80 (0.62, 1.04)
Hospital Level Practices	High Inotropes (A, C, E, G)	2.09 (1.08, 4.05)
	High Vasopressors (E, F, G, H)	0.83 (0.32, 2.14)
	High Transfusions (B, E, G, H)	1.45 (0.62, 3.41)
	High Fluids (D, F, G, H)	1.40 (0.61, 3.19)

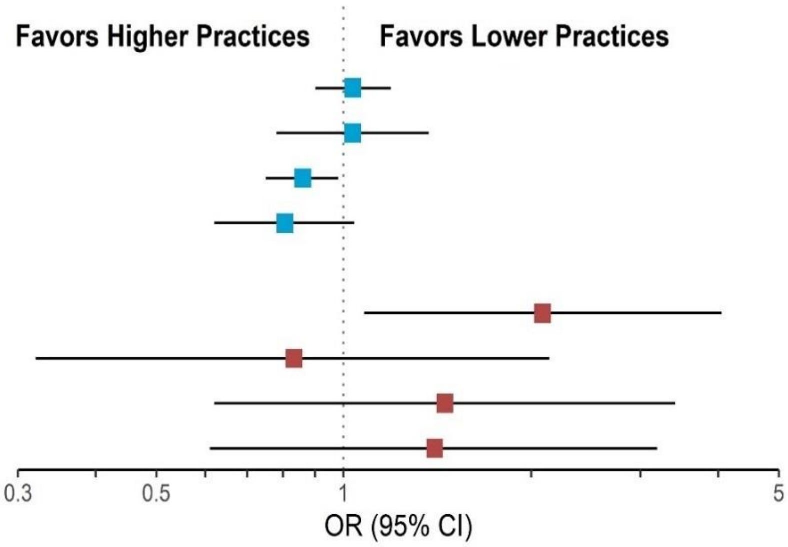

Hospital- and clinician-level operating room practices were defined as high (at or above median) or low (below median) rates of inotrope infusions (epinephrine, milrinone, dobutamine, or dopamine for >60 minutes), vasopressor infusions (phenylephrine, norepinephrine, vasopressin, or angiotensin-II for >60 minutes), homologous red blood cell transfusion, and fluid volume administration (total crystalloid and colloid administered, excluding blood products). Anonymized hospital IDs were provided for the with each "high" practice.

© 2025 Mathis MR et al. *JAMA Network Open*

eFigure 10. Sensitivity Analysis: Forest Plots of Clinician- and Hospital-Level Practices and Adjusted Odds of Acute Kidney Injury, Considering Inopressors Only (Epinephrine, Dopamine)

	Practice	OR (95% CI)
Clinician Level Practices	High Inopressors	1.15 (1.00, 1.31)
Hospital Level Practices	High Inopressors (A, C, E, G)	1.94 (1.15, 3.29)

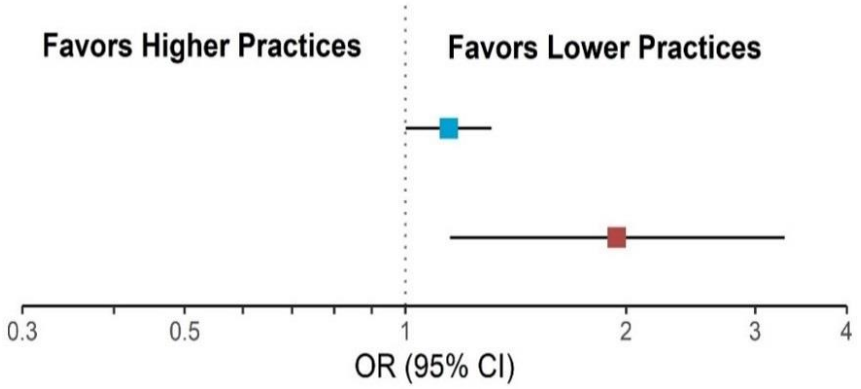

Hospital- and clinician-level operating room practices were defined as high (at or above median) or low (below median) rates of inotrope infusions (epinephrine or dopamine for >60 minutes). Anonymized hospital IDs were provided for the with each "high" practice.

© 2025 Mathis MR et al. *JAMA Network Open*

eFigure 11. Sensitivity Analysis: Forest Plots of Clinician- and Hospital-Level Practices and Adjusted Odds of Acute Kidney Injury, Considering Inotrope and Vasopressor Exposures Based upon Total Intraoperative Infusion Duration

	**Practice**	**OR (95% CI)**
Clinician Level Practices	High Inotropes	1.16 (1.03, 1.29)
	High Vasopressors	0.86 (0.70, 1.05)
Hospital Level Practices	High Inotropes (A, C, E, G)	1.93 (1.15, 3.24)
	High Vasopressors (E, F, G, H)	1.38 (0.62, 3.08)

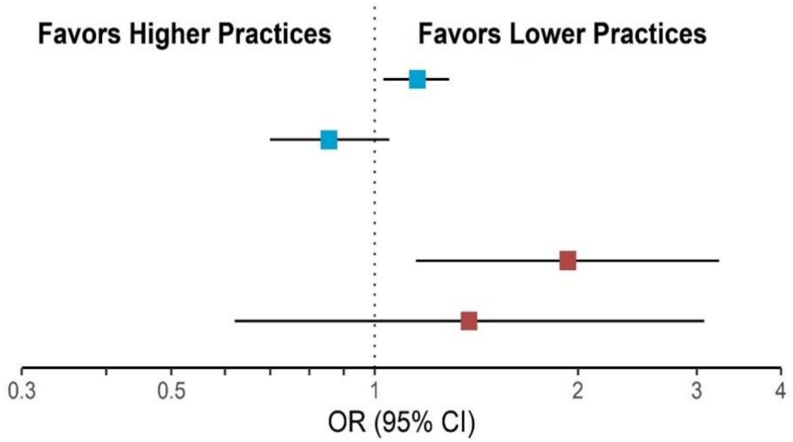

Hospital- and clinician-level operating room practices were defined as high (at or above median) or low (below median) rates of inotrope infusions (epinephrine, milrinone, dobutamine, or dopamine), and vasopressor infusions (phenylephrine, norepinephrine, vasopressin, or angiotensin-II). Anonymized hospital IDs were provided for the with each "high" practice.

© 2025 Mathis MR et al. *JAMA Network Open*
