# Supplementary material for: Hospital and Clinician Practice Variation in Cardiac Surgery and Postoperative Acute Kidney Injury
Source: JAMA Netw Open. 2025 May 2;8(5):e258342. doi: 10.1001/jamanetworkopen.2025.8342 (PMC12048843; doi:10.1001/jamanetworkopen.2025.8342)
Supplement: Supplement 3. — Nonauthor Collaborators [file jamanetwopen-e258342-s003.pdf]

Supplemental Online Content: Nonauthor Collaborators
*First name, last name, and suffix (if applicable) are required and will appear in PubMed.

*Group Name(s): MPOG Collaborators							
***First Name and Middle Initial(s)**	***Last Name**	***Suffix (eg, Jr, III)**	Academic Degrees	Institution	Location (city, state/province, country)	Role or Contribution, eg, chair, principal investigator	Group (if more than 1 Group listed in the byline) and/or Subgroup (eg, Steering Committee)
Thoralf M.	Sundt		MD	Massachusetts General Hospital	Boston, MA, USA	Coordination of local clinical database integration; Acquisition, analysis, or interpretation of data; Critical review of manuscript for important intellectual content	
Michael G.	Fitzsimons		MD	Massachusetts General Hospital	Boston, MA, USA	Coordination of local clinical database integration; Acquisition, analysis, or interpretation of data; Critical review of manuscript for important intellectual content	

Supplemental Online Content: Nonauthor Collaborators

*First name, last name, and suffix (if applicable) are required and will appear in PubMed.

***First Name and Middle Initial(s)**	***Last Name**	***Suffix (eg, Jr, III)**	Academic Degrees	Institution	Location (city, state/province, country)	Role or Contribution, eg, chair, principal investigator	Group (if more than 1 Group listed in the byline) and/or Subgroup (eg, Steering Committee)
Kenneth G.	Shann		CCP	Massachusetts General Hospital	Boston, MA, USA	Coordination of local clinical database integration; Acquisition, analysis, or interpretation of data; Critical review of manuscript for important intellectual content	
Vikram	Kumar		MD, MBBS, MHCM	Massachusetts General Hospital	Boston, MA, USA	Coordination of local clinical database integration; Acquisition, analysis, or interpretation of data; Critical review of manuscript for important intellectual content	

*First name, last name, and suffix (if applicable) are required and will appear in PubMed.

***First Name and Middle Initial(s)**	***Last Name**	***Suffix (eg, Jr, III)**	Academic Degrees	Institution	Location (city, state/province, country)	Role or Contribution, eg, chair, principal investigator	Group (if more than 1 Group listed in the byline) and/or Subgroup (eg, Steering Committee)
Leora T.	Yarboro		MD	University of Virginia School of Medicine	Charleston, VA, USA	Coordination of local clinical database integration; Acquisition, analysis, or interpretation of data; Critical review of manuscript for important intellectual content	
Bhiken I.	Naik		MBBCh, MSCR	University of Virginia School of Medicine	Charleston, VA, USA	Coordination of local clinical database integration; Acquisition, analysis, or interpretation of data; Critical review of manuscript for important intellectual content	

*First name, last name, and suffix (if applicable) are required and will appear in PubMed.

***First Name and Middle Initial(s)**	***Last Name**	***Suffix (eg, Jr, III)**	Academic Degrees	Institution	Location (city, state/province, country)	Role or Contribution, eg, chair, principal investigator	Group (if more than 1 Group listed in the byline) and/or Subgroup (eg, Steering Committee)
Frederick A.	Tibayan		MD	Oregon Health & Science University	Portland, OR, USA	Coordination of local clinical database integration; Acquisition, analysis, or interpretation of data; Critical review of manuscript for important intellectual content	
Michael F.	Aziz		MD	Oregon Health & Science University	Portland, OR, USA	Coordination of local clinical database integration; Acquisition, analysis, or interpretation of data; Critical review of manuscript for important intellectual content	

Supplemental Online Content: Nonauthor Collaborators
*First name, last name, and suffix (if applicable) are required and will appear in PubMed.

***First Name and Middle Initial(s)**	***Last Name**	***Suffix (eg, Jr, III)**	Academic Degrees	Institution	Location (city, state/province, country)	Role or Contribution, eg, chair, principal investigator	Group (if more than 1 Group listed in the byline) and/or Subgroup (eg, Steering Committee)
Michael S.	Mulligan		MD	University of Washington School of Medicine	Seattle, WA, USA	Coordination of local clinical database integration; Acquisition, analysis, or interpretation of data; Critical review of manuscript for important intellectual content	
Srdjan	Jelacic		MD	University of Washington	Seattle, WA, USA	Coordination of local clinical database integration; Acquisition, analysis, or interpretation of data; Critical review of manuscript for important intellectual content	

Supplemental Online Content: Nonauthor Collaborators
*First name, last name, and suffix (if applicable) are required and will appear in PubMed.

***First Name and Middle Initial(s)**	***Last Name**	***Suffix (eg, Jr, III)**	Academic Degrees	Institution	Location (city, state/province, country)	Role or Contribution, eg, chair, principal investigator	Group (if more than 1 Group listed in the byline) and/or Subgroup (eg, Steering Committee)
Tsuyoshi	Kaneko		MD	Washington University School of Medicine in St. Louis	St. Louis, MO, USA	Coordination of local clinical database integration; Acquisition, analysis, or interpretation of data; Critical review of manuscript for important intellectual content	
Thomas J.	Graetz		MD, MBA	Washington University School of Medicine in St. Louis	St. Louis, MO, USA	Coordination of local clinical database integration; Acquisition, analysis, or interpretation of data; Critical review of manuscript for important intellectual content	

*First name, last name, and suffix (if applicable) are required and will appear in PubMed.

***First Name and Middle Initial(s)**	***Last Name**	***Suffix (eg, Jr, III)**	Academic Degrees	Institution	Location (city, state/province, country)	Role or Contribution, eg, chair, principal investigator	Group (if more than 1 Group listed in the byline) and/or Subgroup (eg, Steering Committee)
Roland	Assi		MD, MMS	Yale School of Medicine	New Haven, CT, USA	Coordination of local clinical database integration; Acquisition, analysis, or interpretation of data; Critical review of manuscript for important intellectual content	
Hari R.	Mallidi		MD	Brigham and Women's Hospital, Harvard Medical School	Boston, MA, USA	Coordination of local clinical database integration; Acquisition, analysis, or interpretation of data; Critical review of manuscript for important intellectual content	

Supplemental Online Content: Nonauthor Collaborators
*First name, last name, and suffix (if applicable) are required and will appear in PubMed.

***First Name and Middle Initial(s)**	***Last Name**	***Suffix (eg, Jr, III)**	Academic Degrees	Institution	Location (city, state/province, country)	Role or Contribution, eg, chair, principal investigator	Group (if more than 1 Group listed in the byline) and/or Subgroup (eg, Steering Committee)
Gabriela	Querejeta-Roca		MD	Brigham and Women's Hospital, Harvard Medical School	Boston, MA, USA	Coordination of local clinical database integration; Acquisition, analysis, or interpretation of data; Critical review of manuscript for important intellectual content	
Douglas C.	Shook		MD	Brigham and Women's Hospital, Harvard Medical School	Boston, MA, USA	Coordination of local clinical database integration; Acquisition, analysis, or interpretation of data; Critical review of manuscript for important intellectual content	
Chu-An	Tsai		MS	University of Michigan Medical School	Ann Arbor, MI, USA	Data acquisition and cleaning	
Elizabeth S.	Jewell		MS	University of Michigan Medical School	Ann Arbor, MI, USA	Statistical analysis	

Supplemental Online Content: Nonauthor Collaborators
*First name, last name, and suffix (if applicable) are required and will appear in PubMed.

***First Name and Middle Initial(s)**	***Last Name**	***Suffix (eg, Jr, III)**	Academic Degrees	Institution	Location (city, state/province, country)	Role or Contribution, eg, chair, principal investigator	Group (if more than 1 Group listed in the byline) and/or Subgroup (eg, Steering Committee)
Jonathan P.	Wanderer		MD, MPhil	Vanderbilt University Medical Center	Nashville, TN, USA	Acquisition, analysis, or interpretation of data; Critical review of manuscript for important intellectual content	
Robert E.	Freundlich		MD, MSc	Vanderbilt University Medical Center	Nashville, TN, USA	Acquisition, analysis, or interpretation of data; Critical review of manuscript for important intellectual content	
Peter	Rock		MD, MBA	University of Maryland School of Medicine	Baltimore, MD, USA	Acquisition, analysis, or interpretation of data; Critical review of manuscript for important intellectual content	

Supplemental Online Content: Nonauthor Collaborators
*First name, last name, and suffix (if applicable) are required and will appear in PubMed.

***First Name and Middle Initial(s)**	***Last Name**	***Suffix (eg, Jr, III)**	Academic Degrees	Institution	Location (city, state/province, country)	Role or Contribution, eg, chair, principal investigator	Group (if more than 1 Group listed in the byline) and/or Subgroup (eg, Steering Committee)
Nicholas J.	Douville		MD, PhD	University of Michigan Medical School	Ann Arbor, MI, USA	Acquisition, analysis, or interpretation of data; Critical review of manuscript for important intellectual content	
Clark A.	Fisher		MD, PhD	Yale School of Medicine	New Haven, CT, USA	Acquisition, analysis, or interpretation of data; Critical review of manuscript for important intellectual content	
Nathan L.	Pace		MD, MStat	University of Utah	Salt Lake City, UT, USA	Acquisition, analysis, or interpretation of data; Critical review of manuscript for important intellectual content	

Supplemental Online Content: Nonauthor Collaborators
*First name, last name, and suffix (if applicable) are required and will appear in PubMed.

***First Name and Middle Initial(s)**	***Last Name**	***Suffix (eg, Jr, III)**	Academic Degrees	Institution	Location (city, state/province, country)	Role or Contribution, eg, chair, principal investigator	Group (if more than 1 Group listed in the byline) and/or Subgroup (eg, Steering Committee)
Karen B.	Domino		MD, MPH	University of Washington	Seattle, WA, USA	Acquisition, analysis, or interpretation of data; Critical review of manuscript for important intellectual content	
Gebhard	Wagener		MD	Columbia University Medical Center	New York, NY, USA	Acquisition, analysis, or interpretation of data; Critical review of manuscript for important intellectual content	
Christopher R.	King		MD, PhD	Washington University School of Medicine in St. Louis	St. Louis, MO, USA	Acquisition, analysis, or interpretation of data; Critical review of manuscript for important intellectual content	

Supplemental Online Content: Nonauthor Collaborators
*First name, last name, and suffix (if applicable) are required and will appear in PubMed.

***First Name and Middle Initial(s)**	***Last Name**	***Suffix (eg, Jr, III)**	Academic Degrees	Institution	Location (city, state/province, country)	Role or Contribution, eg, chair, principal investigator	Group (if more than 1 Group listed in the byline) and/or Subgroup (eg, Steering Committee)
Vikas	O'Reilly-Shah		MD, PhD	University of Washington	Seattle, WA, USA	Acquisition, analysis, or interpretation of data; Critical review of manuscript for important intellectual content	
Amit	Bardia		MBBS, MPH	Yale School of Medicine	New Haven, CT, USA	Acquisition, analysis, or interpretation of data; Critical review of manuscript for important intellectual content	
David J.	Clark		MD, PhD	Stanford University Medical Center	Palo Alto, CA, USA	Acquisition, analysis, or interpretation of data; Critical review of manuscript for important intellectual content	
