# Supplementary material for: Hospital and Clinician Practice Variation in Cardiac Surgery and Postoperative Acute Kidney Injury
Source: JAMA Netw Open. 2025 May 2;8(5):e258342. doi: 10.1001/jamanetworkopen.2025.8342 (PMC12048843; doi:10.1001/jamanetworkopen.2025.8342)
Supplement: Supplement 4. — Data Sharing Statement [file jamanetwopen-e258342-s004.pdf]

Data Sharing Statement

Mathis. Hospital and Clinician Practice Variation in Cardiac Surgery and Postoperative Acute Kidney Injury. *JAMA Netw Open*. Published May 02, 2025.
doi:10.1001/jamanetworkopen.2025.8342

Data
Data available: Yes
Data types: Deidentified participant data, Data dictionary
How to access data: mpog-research@med.umich.edu
When available: With publication

Supporting Documents
Document types: Statistical/analytic code
How to access documents: mpog-research@med.umich.edu
When available: With publication

Additional Information
Who can access the data: Researchers whose proposed use of the data has been approved.
Types of analyses: The datasets involved in this study are defined as limited datasets per United States Federal Regulations and require execution of a data use agreement for transfer or use of the data. They are derived from data shared within the Multicenter Perioperative Outcomes Group (MPOG). The investigative team is able to share data securely and transparently conditional on: (i) receipt of a detailed written request identifying the requestor, purpose and proposed use of the shared data, (ii) use of a secure enclave for the sharing of personally identifiable information and (iii) the request is permissible within the confines of existing data use agreements executed between MPOG members.
Mechanisms of data availability: The datasets involved in this study are defined as limited datasets per United States Federal Regulations and require execution of a data use agreement for transfer or use of the data. They are derived from data shared within the Multicenter Perioperative Outcomes Group (MPOG). The investigative team is able to share data securely and transparently conditional on: (i) receipt of a detailed written request identifying the requestor, purpose and proposed use of the shared data, (ii) use of a secure enclave for the sharing of personally identifiable information and (iii) the request is permissible within the confines of existing data use agreements executed between MPOG members.
Any additional restrictions: The datasets involved in this study are defined as limited datasets per United States Federal Regulations and require execution of a data use agreement for transfer or use of the data. They are derived from data shared within the Multicenter Perioperative Outcomes Group (MPOG). The investigative team is able to share data securely and transparently conditional on: (i) receipt of a detailed written request identifying the requestor, purpose and proposed use of the shared data, (ii) use of a secure enclave for the sharing of personally identifiable information and (iii) the request is permissible within the confines of existing data use agreements executed between MPOG members.
